# CircNFATC3 promotes the proliferation of gastric cancer through binding to IGF2BP3 and restricting its ubiquitination to enhance CCND1 mRNA stability

**DOI:** 10.1186/s12967-023-04235-y

**Published:** 2023-06-20

**Authors:** Feifei Yang, Qiang Ma, Bo Huang, Xiaolin Wang, Xiaojuan Pan, Ting Yu, Lingyu Ran, Shan Jiang, Haiping Li, Ye Chen, Yuying Liu, Ce Liang, Junwu Ren, Yuying Zhang, Shimin Wang, Wei Li, Bin Xiao

**Affiliations:** 1 College of Pharmacy, Chongqing Medical University, Chongqing, 400016 People’s Republic of China; 2 Department of Clinical Laboratory, Affiliated Hospital of North Sichuan Medical College, Nanchong, 637000 People’s Republic of China; 3 Department of Clinical Laboratory, The 89th Hospital of The People’s Liberation Army, Weifang, 261000 People’s Republic of China; 4 Department of Kidney, Southwest Hospital, Army Medical University, Chongqing, 400038 People’s Republic of China; 5 Institute for Advanced Study, Shenzhen University, Shenzhen, 518055 People’s Republic of China; 6 Department of Pharmacy, Chongqing University Cancer Hospital, Chongqing, 400030 People’s Republic of China

**Keywords:** circNFATC3, IGF2BP3, TRIM25, CCND1, Gastric cancer

## Abstract

**Background:**

Insulin like growth factor II mRNA binding protein 3 (IGF2BP3) is an RNA binding protein with multiple roles in regulation of gene expression at the post-transcriptional level and is implicated in tumorigenesis and progression of numerous cancers including gastric cancer (GC). Circular RNAs (circRNAs) are a diverse endogenous noncoding RNA population that have important regulatory roles in cancer. However, circRNAs that regulate the expression of IGF2BP3 in GC is largely unknown.

**Methods:**

CircRNAs that bound to IGF2BP3 were screened in GC cells using RNA immunoprecipitation and sequencing (RIP-seq). The identification and localization of circular nuclear factor of activated T cells 3 (circNFATC3) were identified using Sanger sequencing, RNase R assays, qRT-PCR, nuclear-cytoplasmic fractionation and RNA-FISH assays. CircNFATC3 expression in human GC tissues and adjacent normal tissues were measured by qRT-PCR and ISH. The biological role of circNFATC3 in GC was confirmed by in vivo and in vitro experiments. Furthermore, RIP, RNA-FISH/IF, IP and rescue experiments were performed to uncover interactions between circNFATC3, IGF2BP3 and cyclin D1 (CCND1).

**Results:**

We identified a GC-associated circRNA, circNFATC3, that interacted with IGF2BP3. CircNFATC3 was significantly overexpressed in GC tissues and was positively associated with tumor volume. Functionally, the proliferation of GC cells decreased significantly after circNFATC3 knockdown in vivo and in vitro. Mechanistically, circNFATC3 bound to IGF2BP3 in the cytoplasm, which enhanced the stability of IGF2BP3 by preventing ubiquitin E3 ligase TRIM25-mediated ubiquitination, thereby enhancing the regulatory axis of IGF2BP3-CCND1 and promoting CCND1 mRNA stability.

**Conclusions:**

Our findings demonstrate that circNFATC3 promotes GC proliferation by stabilizing IGF2BP3 protein to enhance CCND1 mRNA stability. Therefore, circNFATC3 is a potential novel target for the treatment of GC.

**Supplementary Information:**

The online version contains supplementary material available at 10.1186/s12967-023-04235-y.

## Background

Gastric cancer (GC) is a common malignancy that affects over 1 million people annually with a mortality of 7.7% worldwide [1]. Endoscopic or surgical treatments are effective for early-stage GC but advanced stages GC that presents a high risk of metastasis is often treated with chemotherapies such as platinum and fluoropyrimidine, however, their efficacy is limited [2, 3]. Thus, a more comprehensive knowledge of GC pathogenesis is necessary to identify effective diagnostic and therapeutic targets.

RNA binding proteins (RBPs) are key regulators of RNA metabolism and have been implicated in both cancer initiation and progression [4]. The insulin like growth factor II mRNA binding protein 3 (IGF2BP3) is one of the RBPs that contains 6 RNA-binding domains, including 2 RNA recognition motifs (RRM) at the N-terminus and 4 hnRNPK homology (KH) domains at the C-terminus. Existing studies have shown that IGF2BP3 expression is abnormally elevated in a variety of cancers and is positively associated with tumor malignancy [5–7]. Usually, IGF2BP3 exerts its biological functions in cancers via regulation of mRNA cleavage and stability and through translational regulation [8–11]. Recent studies have shown that IGF2BP3 can interact with non-coding RNA to regulate cancer progression [12–14]. Our previous studies and others have confirmed that IGF2BP3 is an oncogenic factor in GC that promotes proliferation and migration of GC cells [15–18]. Nonetheless, the molecular mechanism underlying the expression regulation of IGF2BP3 is largely unknown.

Circular RNAs (circRNAs) are a class of noncoding RNAs with covalently closed single-stranded loop produced by back-splicing of precursor mRNAs that lack 5' G-caps and 3' A tails [19, 20]. CircRNAs perform important biological functions in various tumors via miRNA sponges, alternative splicing, binding proteins, mRNAs stabilization, and as a template of translation [21–25]. Interestingly, previous studies have demonstrated that some circRNAs can bind to IGF2BP3 protein in GC and affect tumorigenesis and progression [15, 17]. However, whether circRNAs directly regulate IGF2BP3 expression in GC is still unclear.

Cyclin D1 (CCND1) is an effector gene that promotes tumor progression in a variety of cancers [26–28]. In colon cancer, IGF2BP3 increases the stability of CCND1 mRNA and promotes the proliferation of cancer cells [29]. In GC, although IGF2BP3 and CCND1 are both highly expressed in the TCGA database and positively correlated in the TIMER 2.0 database, whether CCND1 is regulated by IGF2BP3 in GC remains unclear.

Here, we identified an IGF2BP3-binding circRNA, circNFATC3, that was upregulated in GC and its expression promoted proliferation of GC cells in vivo and in vitro. Mechanistically, circNFATC3 directly bound to IGF2BP3 and enhanced the stability of IGF2BP3 by preventing tripartite motif-containing 25 (TRIM25)-mediated ubiquitination, thereby promoting Cyclin D1 (CCND1) mRNA stability.

## Methods

### Clinical specimens

GC tissues and corresponding adjacent non-tumour (NC) tissues (16 paired samples) were obtained from Southwest Hospital of the Army Medical University. All patients did not receive any neoadjuvant therapy before operation and were diagnosed as by postoperative pathology. The surgically excised specimens were collected in EP tubes filled with RNA Later (Thermo Scientific, Pittsburg, PA, USA) and stored at − 80 °C. Clinical characteristics are listed in Additional file 1: Table S1. All samples were collected with the informed consent of the patients and the study was approved by the Ethics Review Committee of Chongqing Medical University and Southwest Hospital of the Army Medical University.

### Cell lines

The human GC cell lines SGC7901, BGC823 and HGC-27 cells were obtained from the Army Medical University (Chongqing, China). SGC7901 and BGC823 cells were cultured in Dulbecco’s modified Eagle’s medium (DMEM) with 10% foetal bovine serum (FBS) (Lonsera, Uruguay). HGC-27 cells were cultured in RPMI 1640 medium (Basal Media, Shanghai, China) supplemented with 10% FBS. All cells were cultured at 37 °C in a humidified incubator with 5% CO_2_ (standard incubation conditions).

### RNase R treatment

Total RNA (5 μg) was incubated with or without 6 U RNase R (Lucigen, Middleton, WI, USA) at 37 °C for 10 min, followed by incubation at 85 ℃ for 5 s. After treatment, expression levels of NFATC3 and circNFATC3 were determined using qRT-PCR.

### Actinomycin D assay

CircNFATC3 stability assays utilized GC cells seeded into 12-well plates that were treated with medium containing actinomycin D (5 μg/ml) (Genview, Beijing, China) or DMSO for 0, 3, 6 and 9 h. The cells were then lysed in RNAiso Plus (Takara, Kyoto, Japan) for RNA extraction to assess NFATC3 mRNA and circNFATC3 stability using qRT-PCR. For mRNA stability assays, GC cells separately containing IGF2BP3 knockdown and overexpression and circNFATC3 knockdown systems were cultured in 12-well plates and treated with actinomycin D (5 μg/ml) or DMSO for the indicated times. The cells were lysed in RNAiso Plus for RNA extraction and qRT-PCR measurements.

### Nuclear-cytoplasmic fractionation

RNA from nuclear and cytoplasm fractions of GC cells were separated using a commercial Paris kit (Life Technologies, Gaithersburg, MD, USA) according to the manufacturer’s instructions. CircNFATC3, NFATC3 and GAPDH cDNA were obtained using a PrimeScript RT Master Mix (Takara) and snoU6 cDNA was synthesized by stem-loop methods (RiboBio, Guangzhou, China). The expression levels of circNFATC3, NFATC3, GAPDH and snoU6 in nuclear and cytoplasm were determined by qRT-PCR.

### RNA-fluorescence in situ hybridization (RNA-FISH) and RNA-FISH/IF assay

RNA fluorescence in situ hybridization (FISH) assays were conducted using GC cells seeded in μ-Slide 8 well chamber slide (Ibidi, Martinsried, Germany) that were incubated for 24 h. And then FISH assays were performed using a commercial kit (GenePharma, Shanghai, China) according to the manufacturer’s instructions. Biotin-labeled probes (Additional file 1: Table S2) were synthesized by Genepharma and used to visualize circNFATC3 in situ.

RNA-FISH/IF assays utilized GC cells prepared for FISH as per above with modifications. Briefly, GC cells were incubated with 10% BSA for 30 min at 37 ℃ and subsequently incubated with specific antibody for IGF2BP3 (Abcam, Cambridge, UK) at a 1:400 dilution at 4 °C overnight. The cells were washed for 3 × with PBS and incubated with fluorescent secondary antibody (Signalway Antibody, Nanjing, China) at 1:400 dilution for 1 h at room temperature and washed 3 × with PBS. Images were acquired with a confocal microscope (Leica Microsystems, Wetzlar, Germany).

### RNA extraction and quantitative real-time PCR (qRT-PCR) analysis

Total RNA was extracted using RNAiso Plus (Takara) and then converted into cDNA with a PrimeScript RT Master Mix (Takara). Real-time qPCR was performed using SYBR Green Master Mix (Bioground, Chongqing, China) with CFX connect Real-Time PCR System (BioRad, Hercules, CA, USA). The 2^−ΔΔCt^ method was used to analyze the relative expression levels of indicated genes and GAPDH was used as the normalizing control. The primers are listed in Additional file 1: Table S3.

### Tissue microarray (TMA) and in situ hybridization (ISH)

TMAs were produced from 180 paraffin-embedded samples by Outdo Biotech (Shanghai, China) and clinical characteristics for the sample sources are listed in Additional file 1: Table S4. ISH was performed using a commercial kit (Boster, Wuhan, China) to detect circNFATC3 expression in GC and adjacent tissues using a digoxin-labelled probe against circNFATC3 (Additional file 1: Table S5). Concretely, TMAs were dewaxed in xylene and rehydrated with a 100, 95, 85 and 75% ethanol series. The specimens were treated with a mixture of 30% H_2_O_2_ and distilled water (1:10) at room temperature for 5 min to inactivate endogenous enzymes, and then digested for 2 min at 37 ℃ with a freshly diluted pepsin drops of 3% citric acid. Next, the specimens were washed 3 × with in situ hybridization PBS and again with distilled water. Then the specimens were fixed with fixing solution for 10 min and washed 3 × with distilled water. The digoxin-labelled probe hybridization solution was added to the specimens and pre-hybridized for 2 h at 50 °C, and then hybridized at 50 °C overnight. The specimens were washed with sodium citrate buffer for 40 min at 50 ℃ and then closed with blocking solution for 30 min at 37 ℃. The specimens were incubated with biotin-conjugated anti-digoxin antibody for 1 h at 37 ℃ and then washed 4 × with in situ hybridization PBS. The specimens were reacted with SABC for 20 min at 37 ℃ and washed 3 × with in situ hybridization PBS. The specimens were incubated with biotinylated peroxidase for 20 min at 37 ℃ and washed 4 × with in situ hybridization PBS. And then the specimens stained with NBT/BCIP (Roche) and photographed. CircNFATC3 expression was quantified using staining scores utilized the staining intensity that was ranked as follows: 0, no expression; 1, mildly positive; 2, moderately positive; 3, markedly positive and the scores were then multiplied by numbers of positive stained cells as follows: 0, < 5%; 1, 5–25%; 2, 26–50%; 3, 51–75%; 4, > 75%.

### Cell transfections

Small interfering RNAs (siRNA) were designed and synthesized by GenePharma (Additional file 1: Table S6). The CDS sequences for IGF2BP3 (NM_006547) and CCND1 (NM_053056) were cloned into the pCDH plasmid vector to construct pCDH- IGF2BP3 or pCDH-CCND1 overexpression plasmids. Plasmids carrying the full length of IGF2BP3 or truncations were constructed in the pEGFP-N1 vector (Clontech, Takara). Plasmid pCMV3-N-HA-TRIM25 was purchased from Sino Biological (Beijing, China) (HG17528-NY) and pCMV-HA-Ub plasmids were provided by Professor Lei Liu from the First Affiliated Hospital of Chongqing Medical University, Chongqing, China. Transient transfections were performed with Lipofectamine 2000 reagent (Invitrogen, Carlsbad, CA, USA) or a commercial DNA transfection reagent (Neofect, Beijing, China) according to the manufacturer’s instructions. To construct stable cell lines, pCDH-IGF2BP3 and pCDH-CCND1 plasmids were co-packaged in 293T cells using a commercial lentivirus packaging system with the plasmids pMD2.G, pCMV-VSV-G and pRSV-Rev. Subsequently, GC cells were infected with lentivirus containing IGF2BP3, CCND1 or sh-circNFATC3 (Hanbio, Shanghai, China) and screened by incubation in the presence of 2 μg/ml puromycin.

### Western blotting

Total protein was extracted using RIPA lysis buffer containing PMSF and phosphatase inhibitor (Beyotime, Shanghai, China). The protein samples were separated on SDS-PAGE gels and electrotransferred to polyvinylidene fluoride membranes (PVDF). The membranes were blocked with 5% BSA, and then incubated with primary antibodies overnight at 4 ℃. The membranes were washed with PBST and incubated with HRP-conjugated secondary antibodies (Beyotime) at room temperature for 1 h. The membranes were incubated with ECL chemiluminescent reagent (BioScience) for visualization. The antibodies used in these experiments were as follows: β-actin (Beyotime), IGF2BP3 and TRIM25 (Abcam, Cambridge, UK), CCND1 (CUSabio, Wuhan, China), GFP (Roche Diagnostics GmbH Mannheim, Germany) and HA (Abmart, Shanghai, China).

### RNA-binding protein immunoprecipitation RNA (RIP)

RIP experiments were performed using a Magna RIP kit (Millipore, Burlington, MA, USA). Briefly, cell lysates from GC cells possessing IGF2BP3 or circNFATC3 knockdowns or IGF2BP3 truncations and overexpression plasmids were incubated with RIP buffer containing antibody against IGF2BP3 (Abcam) or GFP (Roche) or control IgG conjugated magnetic beads overnight at 4℃. Subsequently, immunoprecipitated RNA was extracted and purified from the samples and quantitative analysis of the enriched RNA was performed using qRT-PCR.

### RNA pull-down assays

RNA pull-down assays were performed with RNA pull-down kit (Chongqing, China). Briefly, cell lysates were incubated with streptavidin magnetic beads that bound the biotin-labeled circNFATC3 pull-down probe or negative control probe (Additional file 1: Table S7) overnight at 4℃. Protein complexes bound to beads were identified using Western blotting.

### Cell proliferation assays

The CCK-8 kit (Cell Counting Kit-8, Biosharp, Hefei, China) was employed with 3 × 10^3^ cells seeded into a 96-well plate with 100 μl complete culture media and incubated under standard conditions. CCK-8 reagent and complete culture media were added into 96-well plates at 10:100 ratio and the plates were incubated for 2 h under standard conditions and absorbance at 450 nm was measured with a Varioskan LUX microplate reader (Thermo Scientific, Waltham, MA, USA) to measure cell viability.

Plate colony formation assays utilized 1 × 10^3^ cells seeded in 12-well plates containing 1 ml complete culture media that were cultured for 1–2 weeks. The cell spheres were fixed with 4% paraformaldehyde for 10 min and then stained with 1% crystal violet for 10 min. The plates were washed 3 × with water and air-dried at ambient temperature. Images were scanned using an Epson scanner (Suwa, Nagano Prefecture, Japan).

EdU (5-ethynyl-2′-deoxyuridine) assays utilized 3 × 10^4^ cells seeded in 48-well plates containing 250 μl complete culture media and cultured for 24 h. A commercial EdU kit (Ribobio) was used to measure cell proliferation ability according to the manufacturer’s instructions. Cells were photographed with a fluorescent inverted microscope (Olympus, Tokyo, Japan).

### Animal experiments

Male BALB/c nude mice (GemPharmatech, Chengdu, China) aged 3–4 weeks were employed for our in vivo study. To construct subcutaneous xenografts, 5 × 10^6^ HGC-27 cells were suspended in 100 μl PBS and injected subcutaneously on the backs of BALB/c nude mice. One week later, the size of the xenograft tumor was measured with a caliper and for every 2 days thereafter. Tumor volumes were calculated using the formula: length × width^2^/2. After the xenograft tumor grew to an appropriate volume, the nude mice were randomly divided into groups. To study the proliferative function of circNFATC3 in vivo, cholesterol modified siRNA targeting circNFATC3 or negative control (Genepharma, 5 nmol/kg) was dissolved in 25 μl sterile physiological saline and mixed with 5 μl in vivo transfection reagent (Engreen, Beijing, China) and then injected into the tumor each 2 days for 2–3 weeks. In vivo rescue assays utilized tumor- bearing nude mice treated with a cholesterol-modified siRNA targeting circNFATC3 or negative control combined with lentivirus containing CCND1 or pCDH (Leqin Biotechnology, Chongqing, China) using siRNA levels as per above. The lentivirus containing CCND1 was also injected into the tumor mass once a week for 2 weeks. The animals were sacrificed when the tumor size of the nude mice was appropriate. All animal studies were approved by the Ethics Review Committee of Chongqing Medical University.

### Immunohistochemistry (IHC) staining

IHC was performed as previously described [18]. The primary antibodies used in the experiments included anti-Ki-67 (1:500, Servicebio, Wuhan, China), anti-IGF2BP3 (1:50, Abcam) and anti-CCND1 (1:100, CUSabio). Sections were photographed with an optical microscope (Leica Microsystems, Germany) and 3 different visual fields were randomly captured. Protein staining scores on tissue sections were calculated as follows: 0, < 5%; 1, 5–25%; 2, 26–50%; 3, 51–75%; 4, > 75% × intensity score; 0, no expression; 1, mild positive; 2, moderate positive; 3, significant positive.

### Cycloheximide (CHX) and proteasome inhibitor (MG132) assays

CHX is an inhibitor of protein synthesis in eukaryotes and is used to detect protein stability. GC cells were transfected with siRNA targeting circNFATC3 using Lipofectamine 2000 (Invitrogen) for 40 h and then treated with CHX (20 µg/ml) (Genview, Beijing, China) for 0, 6 and 12 h. MG132 can effectively reduce the activity of 26S proteasome complex towards ubiquitin binding proteins. GC cells were transfected with si-circNFATC3 for 30 h, and then treated with MG132 (10 µM) (MedChemExpress, New Jersey, USA) for 12 h. IGF2BP3 protein expression was determined using Western blotting.

### Immunoprecipitation (IP) analysis

IP analysis was performed using a commercial Kit (Thermo Scientific, Pittsburg, PA, USA). Briefly, cells were lysed with IP lysis/wash buffer containing PMSF and phosphatase inhibitor (Beyotime). Lysates were incubated with magnetic beads cross-linked with IGF2BP3 (Abcam), HA (Abmart), GFP (Roche) or IgG antibodies overnight at 4 ℃. Proteins enriched by magnetic beads were eluted with elution buffer and identified using Western blotting.

### RNA-sequencing

siRNAs targeting IGF2BP3 were transfected into SGC7901 cells using Lipofectamine 2000 (Invitrogen) according to the manufacturer's instructions. The transfected SGC7901 cells were lysed with Trizol (Takara) and subjected to RNA sequencing by Sinotech Genomics (Shanghai, China).

### Statistical analysis

Statistical analysis was performed using Prism 8 (GraphPad, San Diego, CA, USA). Student’s t tests were used to evaluate differences of continuous variables between the two groups. One-way ANOVA was used to compare the differences of continuous variable among multiple groups. The Mann–Whitney U test was used for ISH and IHC score analyses. *P* values < 0.05 was considered as significant.

## Results

### CircNFATC3, an IGF2BP3-related circRNA, is upregulated in GC

**Fig. 1:**
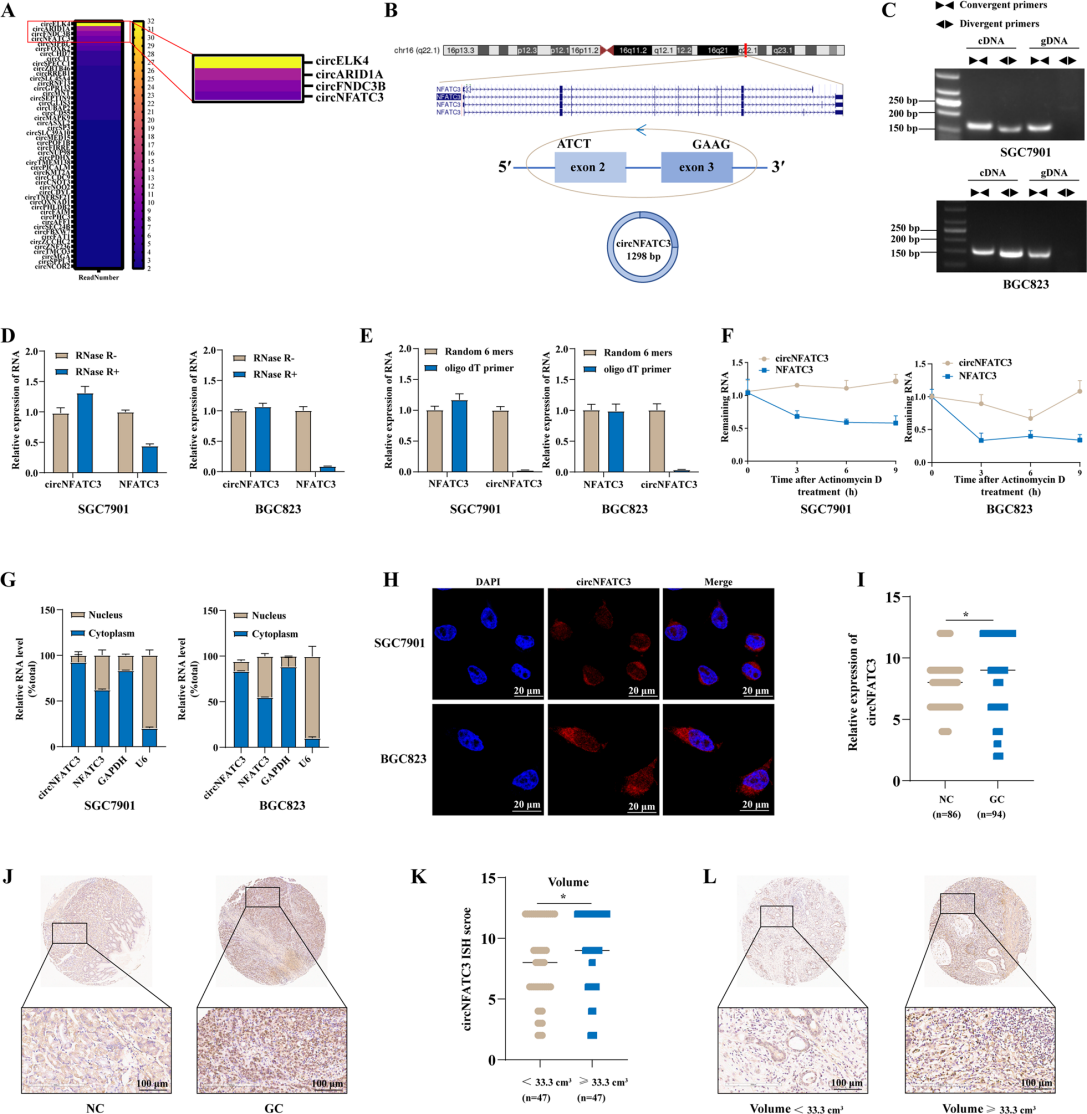
CircNFATC3, an IGF2BP3-related circRNA is upregulated in GC. **A** CircRNAs that bind to IGF2BP3 in SGC7901 cells. **B** Schematic illustration of circNFATC3 formation from its parental gene NFATC3. **C** Existence of circNFATC3 in cDNA or genomic DNA (gDNA) of SGC7901 and BGC823 cells. **D** Expression of circNFATC3 and NFATC3 amplified from total RNA of SGC7901 and BGC823 cells treated with RNase R. **E** Expression of circNFATC3 and NFATC3 amplified from total RNA of SGC7901 and BGC823 cells reverse transcribed by random 6 mers or oligo dT 18 primers.** F** Stabilities of circNFATC3 and NFATC3 in SGC7901 and BGC823 cells treated with actinomycin D. **G** qRT-PCR for the distribution of circNFATC3, NFATC3, GAPDH, and U6 in the cytoplasmic and nuclear fractions of SGC7901 and BGC823.** H** RNA-FISH for circNFATC3 in SGC7901 and BGC823 cells. CircNFATC3 was shown in red and nuclei were stained with DAPI. **I** ISH analysis of circNFATC3 expression in TMA of GC patients (adjacent tissues (NC) = 86 cases; GC tissues = 94 cases).** J** Representative images of circNFATC3 staining in GC and adjacent tissues (NC). **K** Expression of circNFATC3 in GC patients with tumor volumes < 33.3 cm^3^ or ≥ 33.3 cm^3^.** L** Representative images of circNFATC3 staining in tumor tissues of GC patients with tumor volumes < 33.3 cm^3^ or ≥ 33.3 cm^3^. The Mann–Whitney U test was used to calculate p-values. *P < 0.05

We initially employed RIP-seq assays to identify circRNAs that bound to IGF2BP3 in GC cells. We found > 50 circRNAs that interacted with the IGF2BP3 protein and circELK4, circARID1A, circFNDC3B and circNFATC3 displayed the greatest levels of binding (Fig. 1A and Additional file 1: Table S8). 16 paired GC and adjacent tissues from GC patients were used to screen for these circRNAs, and we found that circARID1A, circFNDC3B and circNFATC3 were upregulated in GC tissues compared with adjacent tissues while circELK4 was not (Additional file 2: Fig. S1). However, links to GC for circARID1A and circFNDC3B were identified by us and other laboratories in previous studies [17, 18]. We therefore focused on the circNFATC3, which was not reported in GC, and further evaluated its interactions with the IGF2BP3 protein.

CircNFATC3 (CircBase ID: Hsa_circ_0000711) was composed of exons 2 and 3 of NFATC3 gene (Fig. 1B) and further confirmed by Sanger sequencing for tissue samples as well as in GC cell lines (Additional file 2: Fig. S2). Moreover, circNFATC3 could only be amplified from cDNA using divergent primers and no specific amplification products were observed using genomic DNA templates. In contrast, the linear RNA of NFATC3 was amplified from both cDNA and genomic DNA by convergent primers (Fig. 1C). Furthermore, circNFATC3 expression was not significantly altered in SGC7901 and BGC823 cells after treatment with RNase R indicating an intact circular structure, while the expression of linear NFATC3 was significantly downregulated in the 2 GC cell lines (Fig. 1D). CircNFATC3 was only amplified from cDNA that had been reverse transcribed using random 6-mers while the linear NFATC3 could be amplified using either 6-mers and oligo dT (Fig. 1E). The T_1/2_ of circNFATC3 was also greater than linear NFATC3 in SGC7901 and BGC823 cells that had been treated with actinomycin D (Fig. 1F). CircRNA’s functions are tightly linked to cellular location and we found that circNFATC3 was predominantly localized to the cytoplasm of GC cells (Fig. 1G–H). We also employed tissue microarrays containing 94 GC tissues and 86 peritumor tissues to evaluate the expression of circNFATC3 in human GC. We found that circNFATC3 was significantly upregulated in GC tissues compared with that in the adjacent tissues (Fig. 1I, J) and positively linked to tumor volume (Fig. 1K, L). Taken together, these results demonstrated that circNFATC3 was a circRNA and upregulated in GC tissues.

### CircNFATC3 directly binds to IGF2BP3 protein in GC

**Fig. 2:**
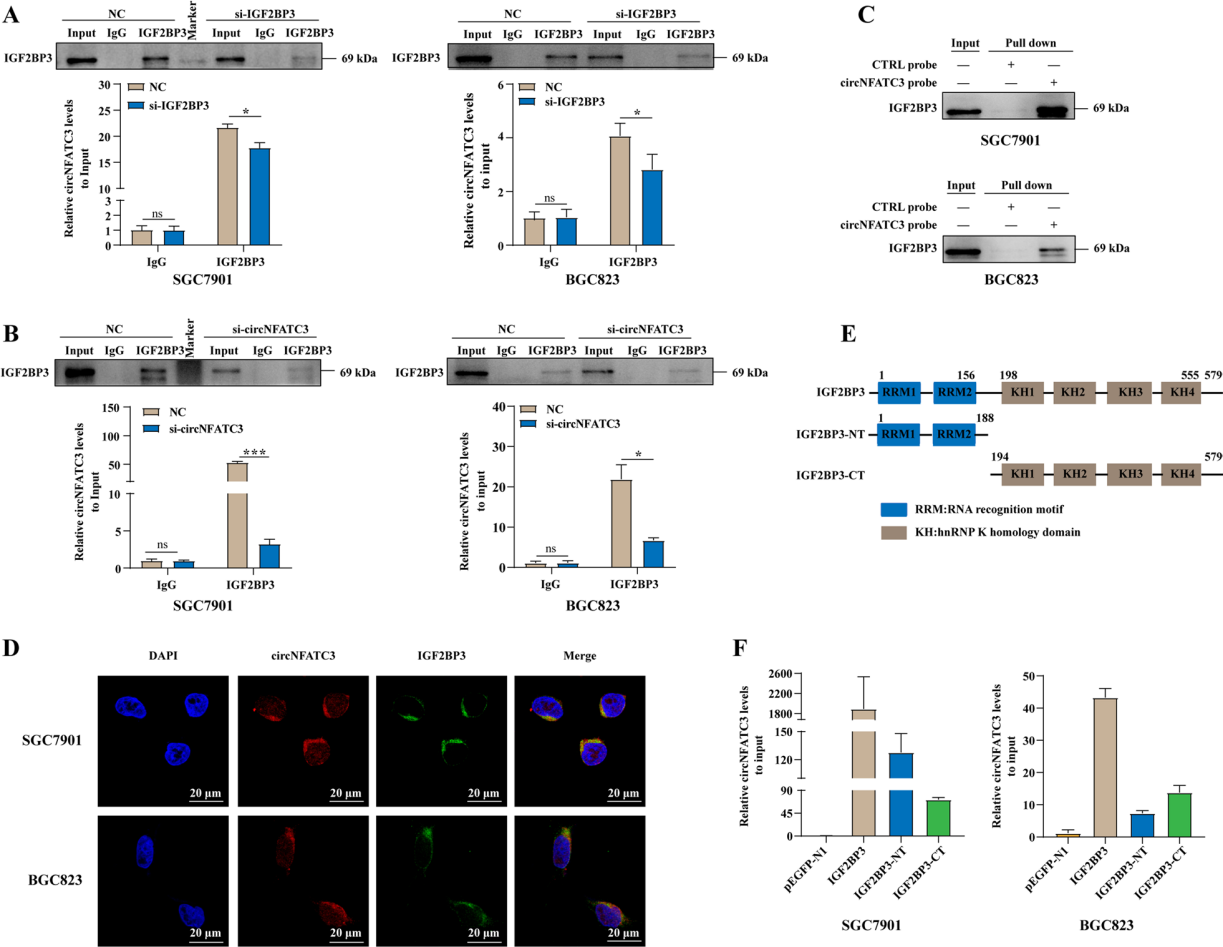
CircNFATC3 directly binds to IGF2BP3 protein in GC. **A-B** RIP analyses of circNFATC3 enrichment pull-downs by IGF2BP3 antibodies in SGC7901and BGC823 cells following IGF2BP3 (**A**) or circNFATC3 (**B**) knockdown. **C** RNA pull-down validation of the interaction between circNFATC3 and IGF2BP3 protein in SGC7901and BGC823 cells. **D** RNA-FISH-immunofluorescence showing the co-localization of circNFATC3 (red) with IGF2BP3 (green) in SGC7901 and BGC823 cells. **E** Structural diagram of IGF2BP3 protein and IGF2BP3 truncations.** F** RIP analysis of circNFATC3 enrichment pulled down by GFP antibodies in SGC7901 and BGC823 cells transfected with full-length or IGF2BP3 truncations. The Student’s t test was used to calculate p-values. ns, not significant, *P < 0.05, ***P < 0.001

For further validation of circNFATC3-IGF2BP3 interaction, we firstly designed two siRNAs targeting circNFATC3(si-circNFATC3-1 and si-circNFATC3-2) and evaluated the expression of circNFATC3 and linear NFATC3 after circNFATC3 knockdowns in GC cells. We found that the both siRNAs targeting circNFATC3 inhibited expression of circNFATC3 in both SGC7901 and BGC823 cells while no detectable change of NFATC3 expression was observed (Additional file 2: Fig. S3A, B). The better inhibition efficiency siRNA si-circNFATC3-2 (also called si-circNFATC3) was then chosen for subsequent functional studies. RIP assays were employed to evaluate circNFATC3 and IGF2BP3 protein binding. We found that circNFATC3 enrichment on IGF2BP3 in GC cells was significantly decreased in SGC7901 and BGC823 cells after IGF2BP3 or circNFATC3 knockdowns (Fig. 2A, B and Additional file 2: Fig. S4A, B). Moreover, RNA pull-down assays demonstrated that the circNFATC3 probe but not the control probe, could enrich for the IGF2BP3 protein in SGC7901 and BGC823 cells (Fig. 2C). Furthermore, co-localization signals for circNFATC3 and IGF2BP3 were observed in the cytoplasm of these cells (Fig. 2D). We additionally constructed IGF2BP3 truncations to evaluate the domains that interacted with circNFATC3. RIP assays indicated that both the N and C termini of IGF2BP3 could interact with circNFATC3 in SGC7901 and BGC823 cells (Fig. 2E, F). Together, these results revealed that circNFATC3 bound to IGF2BP3 protein in GC cells.

### Knockdown of circNFATC3 inhibits the proliferation of GC cells in vitro and in vivo

**Fig. 3:**
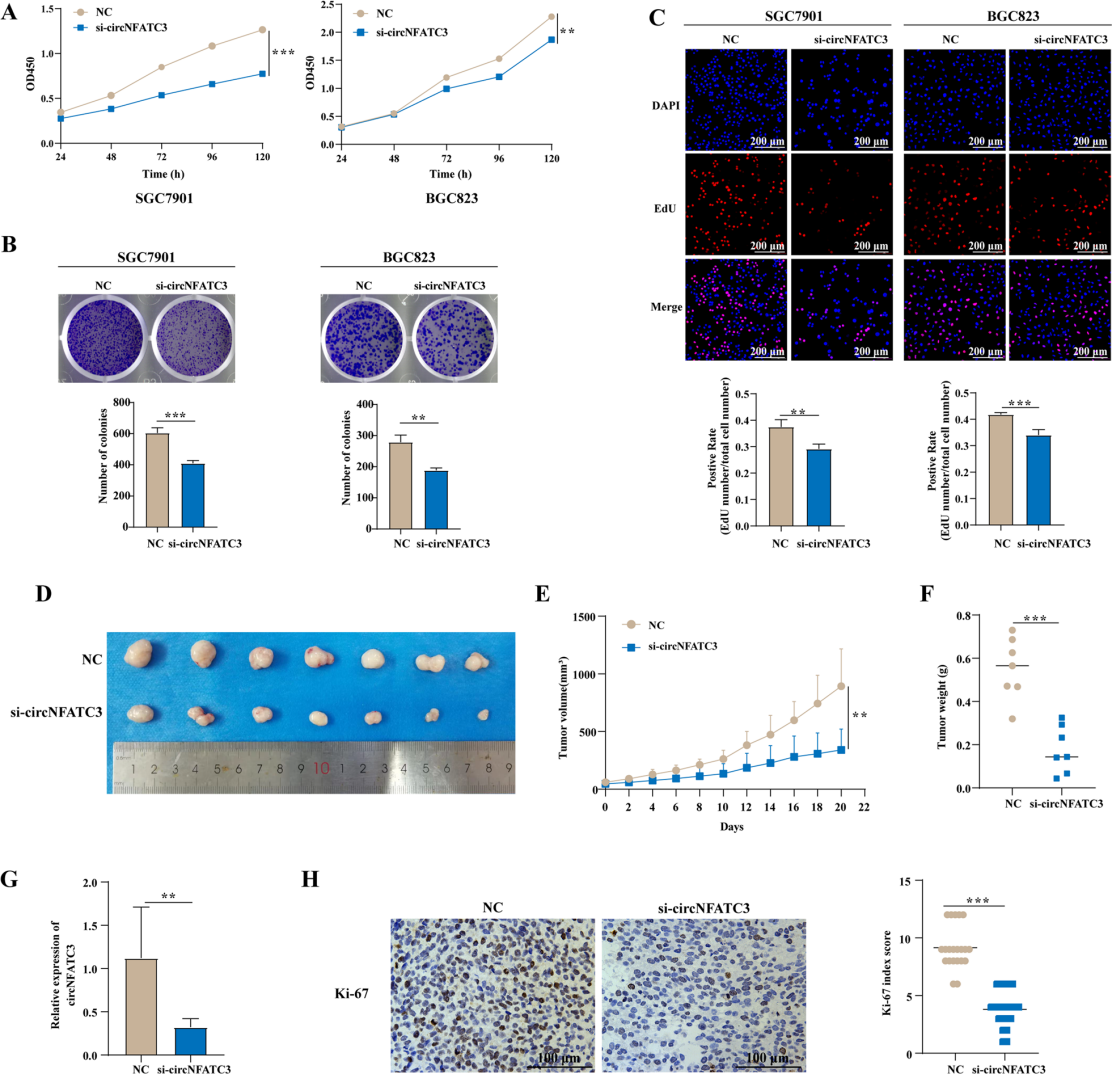
Knockdown of circNFATC3 inhibits the proliferation of GC cells in vitro and in vivo. **A-C** The proliferation of GC cells transfected with circNFATC3 siRNAs and evaluated by CCK-8 (**A**), plate colony formation (**B**), and EdU assays (**C**). **D-F** Tumor images (**D**), growth curves (**E**), and tumor weights (**F**) were obtained from xenograft tumor derived from HGC-27 cells which treated with cholesterol modified siRNAs targeting circNFATC3. **G** Relative expression of circNFATC3 in xenografted tumor tissues. **H** Representative images of Ki-67 expression evaluated by IHC in xenografted tumor tissues. Three different visual fields were randomly selected for each section for staining scoring. The Student’s t test (**A-C, E–G**) and Mann–Whitney U test (**H**) were used to calculate p-values. ** P < 0.01, and *** P < 0.001

We next explored the biological role of circNFATC3 in GC. We found that both si-circNFATC3-1 and si-circNFATC3-2 significantly inhibited SGC7901 and BGC823 cell viability (Fig. 3A and Additional file 2: Fig. S5A). Moreover, proliferation of these cells was also suppressed after knockdown of circNFATC3 as assessed using plate colony formation and EdU assays (Fig. 3B, C and Additional file 2: Fig. S5B, C). Subsequently, we constructed SGC7901 cells with circNFATC3 stable knockdown by lentivirus containing shRNA targeting circNFATC3 (Additional file 2: Fig. S6A), we found that proliferation of these cells was inhibited in accordance with the function of si-NFATC3 (Additional file 2: Fig. S6B–D). These results demonstrated that circNFATC3 might promote the proliferation of GC in vitro. Ideally, the biological role of circNFATC3 in GC evaluated by siRNA should be validated using circNFATC3 overexpression. We therefore utilized a series of circNFATC3 overexpression plasmids constructed by cloning the full-length circNFATC3 in the commercial expression plasmids pLC5-ciR, pcDNA3.1(+) circRNA mini vector and pCD-ciR. However, we found no significant overexpression of circNFATC3 following transfection of these cloned constructs, while the overexpression plasmids constructed by pcDNA3.1(+) CircRNA Mini Vector and pCD-ciR could transcribe linear product of circNFATC3 (Additional file 2: Fig. S7A–F). These results demonstrated that the circNFATC3 sequence did not readily circularize using these in vitro conditions for unknown reasons. To exclude the influence of circRNA sequence on cyclization, we generated another 2 circRNAs (circPDHK1 and circTNPO3) that were inserted into pLC5-ciR or pCD-ciR and the resulting recombinant plasmids (pLC5-ciR-circPDHK1 and pCD-ciR-circTNPO3) were transfected into SGC901 and BGC823 cells. We found that both circPDHK1 and circTNPO3 were effectively overexpressed (Additional file 2: Fig. S7G, H).

We further investigated the function of circNFATC3 in vivo by implanting HGC-27 cells subcutaneously into BALB/c nude mice followed by cholesterol modified si-circNFATC3 injections into the tumors every 2 days for 3 weeks after subcutaneous tumorigenesis. We found that si-circNFATC3 resulted in significant reduction of tumor size and tumor weight (Fig. 3D–F). In addition, the expression of circNFATC3 and Ki-67 were downregulated in these cholesterol-modified si-circNFATC3-treated tumors (Fig. 3G, H). Taken together, these results revealed that knockdown of circNFATC3 can inhibit the proliferation of GC cells in vitro and in vivo.

### CircNFATC3 enhances the stability of IGF2BP3 by preventing TRIM25-mediated ubiquitination

**Fig. 4:**
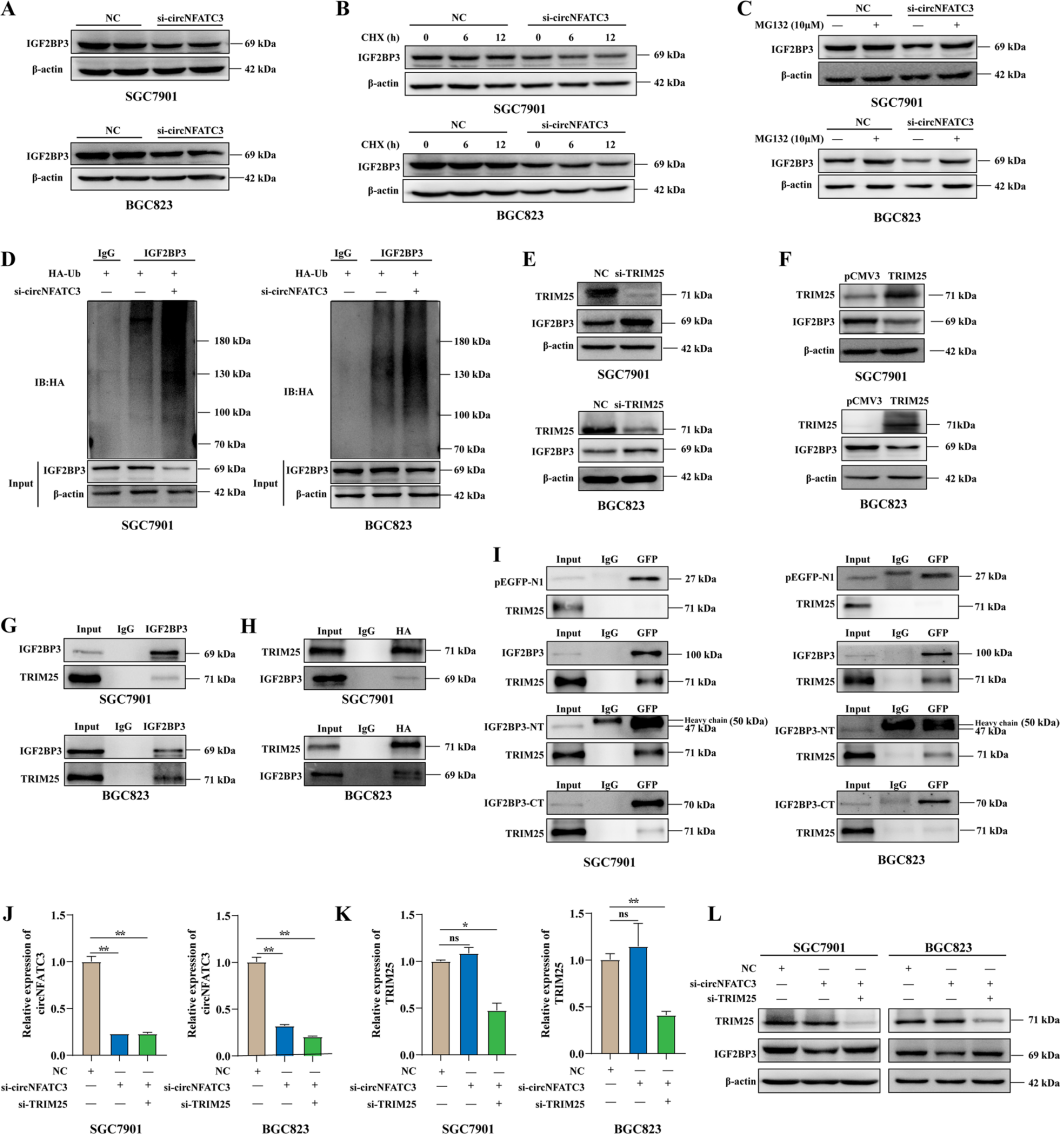
CircNFATC3 enhances the stability of IGF2BP3 by preventing TRIM25-mediated ubiquitination. **A** Western blot analysis of IGF2BP3 in SGC7901 and BGC823 cells transfected with siRNAs targeting circNFATC3. **B** Stability of IGF2BP3 protein in SGC7901 and BGC823 cells after circNFATC3 knockdown evaluated by cycloheximide (CHX) chase assays. **C** Protein expression of IGF2BP3 in SGC7901 and BGC823 cells treated with proteasome inhibitor MG132 after transfection with si-circNFATC3.** D** Immunoprecipitation analysis for ubiquitination modification of IGF2BP3 in SGC7901 and BGC823 cells with circNFATC3 knockdown. **E–F** Protein expression of IGF2BP3 in SGC7901 and BGC823 cells after TRIM25 knockdown (**E)** or overexpression(**F)**. **G-H** Immunoprecipitation analyses of TRIM25 pulled down by IGF2BP3 (**G**) and IGF2BP3 pulled down by TRIM25 (HA-tagged) (**H**) in SGC7901 and BGC823 cells. **I** Immunoprecipitation analysis of TRIM25 pulled down by GFP in SGC7901 and BGC823 cells transfected with full-length of IGF2BP3 or truncations.** J-K** The expression of circNFATC3 (**J**) or TRIM25 (**K**) in SGC7901 and BGC823 cells transfected with si-TRIM25 and/or si-circNFATC3. **L** Protein levels for IGF2BP3 in SGC7901 and BGC823 cells transfected with si-TRIM25 or si-circNFATC3 alone and in combination. The Student’s t test was used to calculate p-values. ns, not significant, *P < 0.05, **P < 0.01

We have demonstrated that circNFATC3 binds to IGF2BP3 but this does not prove a functional regulatory interaction. We therefore used siRNAs to inhibit IGF2BP3 expression in SGC7901 and BGC823 cells (Additional file 2: Fig. S8A, B) and found that circNFATC3 expression was not altered using these constructs (Additional file 2: Fig. S8C). Moreover, circNFATC3 was not significantly altered following IGF2BP3 overexpression in SGC7901 and BGC823 cells (Additional file 2: Fig. S8D–F). Interestingly, we found that IGF2BP3 expression at the protein level but not the mRNA level was significantly downregulated in circNFATC3 knockdown cells (Fig. 4A and Additional file 2: Fig. S8G). These results revealed that circNFATC3 might post-transcriptionally regulate IGF2BP3 expression. We therefore treated SGC7901 and BGC823 cells with CHX combined with the si-circNFATC3 to evaluate the stability of the IGF2BP3 protein after circNFATC3 knockdown. We found that the stability of the IGF2BP3 protein was obviously decreased after circNFATC3 knockdown in both cell lines (Fig. 4B and Additional file 2: Fig. S9). This implicated circNFATC3 might be involved in IGF2BP3 protein degradation in GC.

The autophagy-lysosome and ubiquitin–proteasome pathways are the two primary mechanisms for protein degradation in cells. We therefore evaluated whether the circNFATC3 knockdown induced autophagy in GC cells. The autophagy marker LC3 was not significantly altered following the circNFATC3 knockdown (Additional file 2: Fig. S10). In contrast, the proteasome inhibitor MG132 could partially reverse the decrease of IGF2BP3 protein induced by circNFATC3 knockdown in SGC7901 and BGC823 cells (Fig. 4C). These results revealed that knockdown of circNFATC3 in GC cells induced IGF2BP3 degradation through the proteasome pathway. We subsequently examined whether IGF2BP3 was modified by ubiquitin following the circNFATC3 knockdown. The ubiquitin-modified IGF2BP3 protein was significantly enhanced in cells treated with si-circNFATC3 (Fig. 4D). Since TRIM25 was an E3 ubiquitin ligase for IGF2BP3 [30], we evaluated whether TRIM25 regulated IGF2BP3 expression in GC cells. We found that knockdown of TRIM25 significantly enhanced IGF2BP3 expression (Fig. 4E) while IGF2BP3 protein levels were significantly decreased following TRIM25 overexpression (Fig. 4F). Moreover, IGF2BP3 and TRIM25 were found to be interacting partners as assessed by IP (Fig. 4G, H) and IGF2BP3 truncations indicated that both the N- and C-termini directly interacted with TRIM25 (Fig. 4I and Additional file 2: Fig. S11). Additionally, we knocked-down circNFATC3 and/or TRIM25 in SGC7901 and BGC823 cells (Fig. 4J–K). We found that knockdown of TRIM25 could rescue the downregulation of IGF2BP3 caused by circNFATC3 knockdown (Fig. 4L). Taken together, these data indicated that circNFATC3 interacted with the IGF2BP3 protein and inhibited the degradation of IGF2BP3 trough ubiquitin–proteasome pathway.

### Overexpression of IGF2BP3 can attenuate the proliferation inhibition caused by circNFATC3 knockdown

**Fig. 5:**
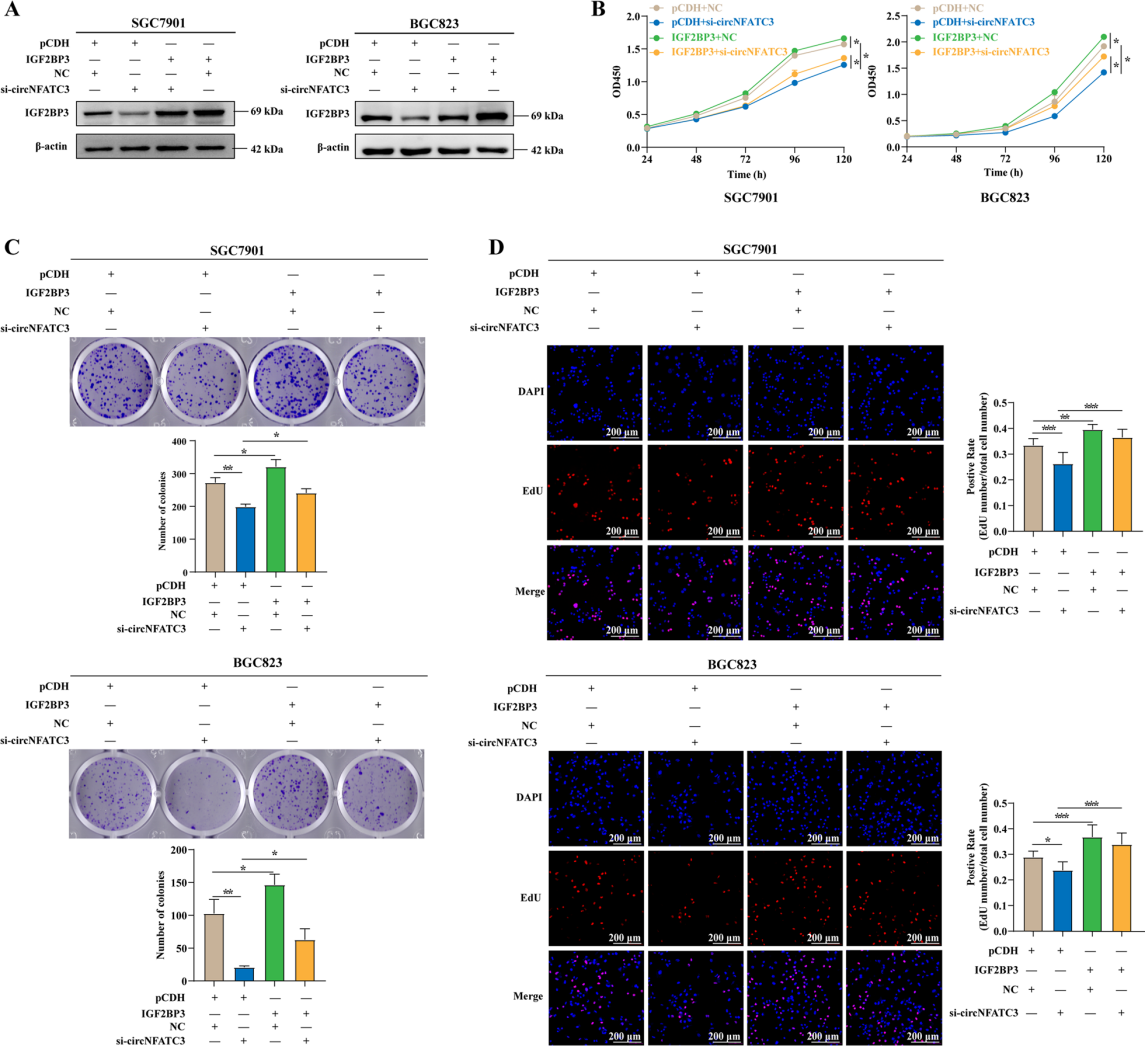
Overexpression of IGF2BP3 can attenuate the proliferation inhibition caused by circNFATC3 knockdown. **A** Protein expression of IGF2BP3 in SGC7901 and BGC823 cells transfected with blank vector or IGF2BP3 overexpression plasmids and co-transfected with NC or si-circNFATC3. **B-D** CCK-8 (**B**), plate colony formation (**C**), and EdU assays (**D**) of SGC7901 and BGC823 cells transfected with blank vector or IGF2BP3 overexpression plasmids and co-transfected with NC or si-circNFATC3 as indicated. One-way ANOVA was used to calculate p-values. *P < 0.05, **P < 0.01, ***P < 0.001

Our results demonstrated that circNFATC3 bound to IGF2BP3 and regulated the expression of IGF2BP3 at the protein level while our previous study confirmed that IGF2BP3 promoted GC cell proliferation [18]. We therefore examined whether the circNFATC3-IGF2BP3 axis influenced the proliferation of SGC7901 and BGC823 cells. We found that overexpression of IGF2BP3 could attenuate the decrease of IGF2BP3 protein caused by a circNFATC3 knockdown (Fig. 5A). Moreover, the inhibition of viability and proliferation in SGC7901 and BGC823 cells caused by the circNFATC3 knockdown was reversed to some extent with IGF2BP3 overexpression (Fig. 5B–D). These data suggested that the circNFATC3 and IGF2BP3 interaction might be the reason for the proliferation-promoting ability of circNFATC3 in GC.

### CircNFATC3 binds to IGF2BP3 to regulate the expression of CCND1 in GC

**Fig. 6:**
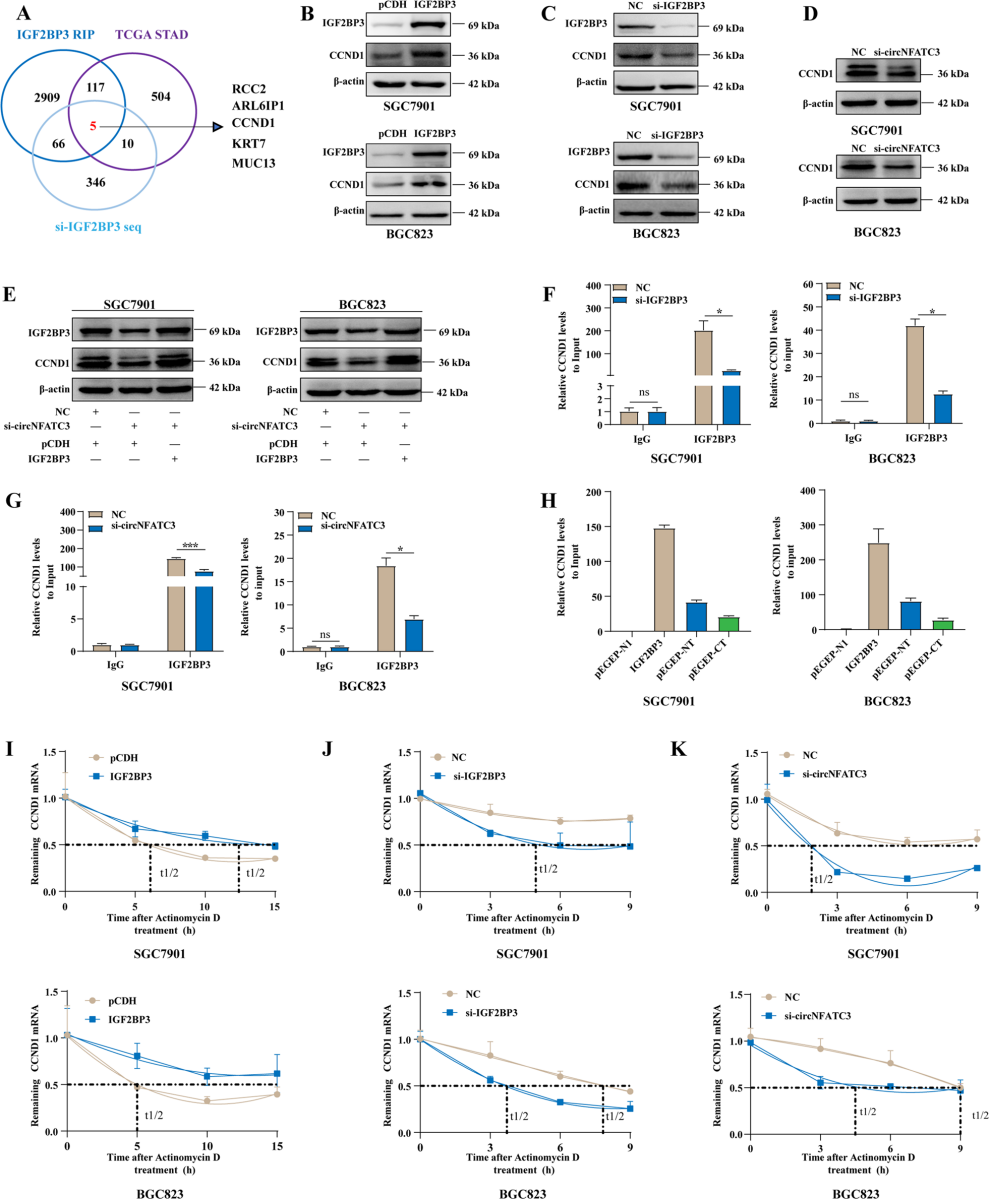
CircNFATC3 binds to IGF2BP3 to regulate the expression of CCND1 in GC. **A** Potential target screening strategy of the circNFATC3-IGF2BP3 axis. **B-D** The protein levels of CCND1 in SGC7901 and BGC823 cells following IGF2BP3 overexpression (**B**) or knockdown (**C**) or circNFATC3 knockdown (**D**). **E** Protein expression of CCND1 in SGC7901 and BGC823 cells transfected with NC, si-circNFATC3 or si-circNFATC3 combined with IGF2BP3 overexpression. **F-G** RIP analyses of CCND1 enrichment pull-downs by IGF2BP3 in SGC7901 and BGC823 cells following IGF2BP3 (**F**) and circNFATC3 (**G**) knockdowns.** H** RIP analysis of CCND1 enrichment pulled down by GFP in SGC7901 and BGC823 cells transfected with full-length or IGF2BP3 truncations. **I-K** Stability of CCND1 mRNA at indicated times following IGF2BP3 overexpression (**I**) or knockdown (**J**) or circNFATC3 knockdown (**K**) in SGC7901and BGC823 cells. The Student’s t test was used to calculate p-values. ns, not significant, *P < 0.05, ***P < 0.001

To develop a more comprehensive understanding of IGF2BP3 and circNFATC3 in GC, we used overlapping genes of three datasets that included the upregulated genes in STAD of the TCGA database, the mRNAs that binding to IGF2BP3 in SGC7901 cells from RIP-seq data and downregulated mRNAs in SGC7901 cells with IGF2BP3 knockdown (Fig. 6A and Additional file 1: Tables S9, S10 and S11). We identified RCC2, ARL6IP1, CCND1, KRT7 and MUC13 as potential target genes of circNFATC3 and IGF2BP3. We validated the expression of these genes in GC cells after IGF2BP3 knockdown, overexpression and circNFATC3 knockdown. We found that only the mRNA level of CCND1 was consistently changed in SGC7901 and BGC823 cells treated with si-IGF2BP3, IGF2BP3 overexpression vector or si-circNFATC3 (Additional file 2: Fig. S12). Furthermore, CCND1 protein levels were upregulated or downregulated in SGC7901 and BGC823 cells treated with IGF2BP3 overexpression or knockdown respectively (Fig. 6B, C). Additionally, circNFATC3 knockdown significantly inhibited CCND1 protein expression (Fig. 6D). These results demonstrated that CCND1 was a likely candidate for a target gene regulated by IGF2BP3 and circNFATC3. We next evaluated whether the interactions between circNFATC3 and IGF2BP3 influenced the expression of CCND1. We found that overexpression of IGF2BP3 partially reversed the down-regulation of CCND1 protein expression caused by interference with circNFATC3 (Fig. 6E). These data indicated that circNFATC3 regulates the IGF2BP3-CCND1 axis.

We additionally found that CCND1 mRNA binding to the IGF2BP3 protein was significantly decreased in SGC7901 and BGC823 cells following IGF2BP3 or circNFATC3 knockdowns (Fig. 6F, G) and both the N- and C-termini of IGF2BP3 could bind CCND1 mRNA (Fig. 6H). Additionally, CCND1 mRNA stability was elevated in cells over-expressing IGF2BP3 (Fig. 6I) but was decreased following knockdown of IGF2BP3 or circNFATC3 (Fig. 6J, K). Taken together, these results demonstrated that circNFATC3 and IGF2BP3 enhanced the expression of CCND1 by regulating stability of its mRNA.

### CircNFATC3 promotes the proliferation of GC via CCND1 regulation

**Fig. 7:**
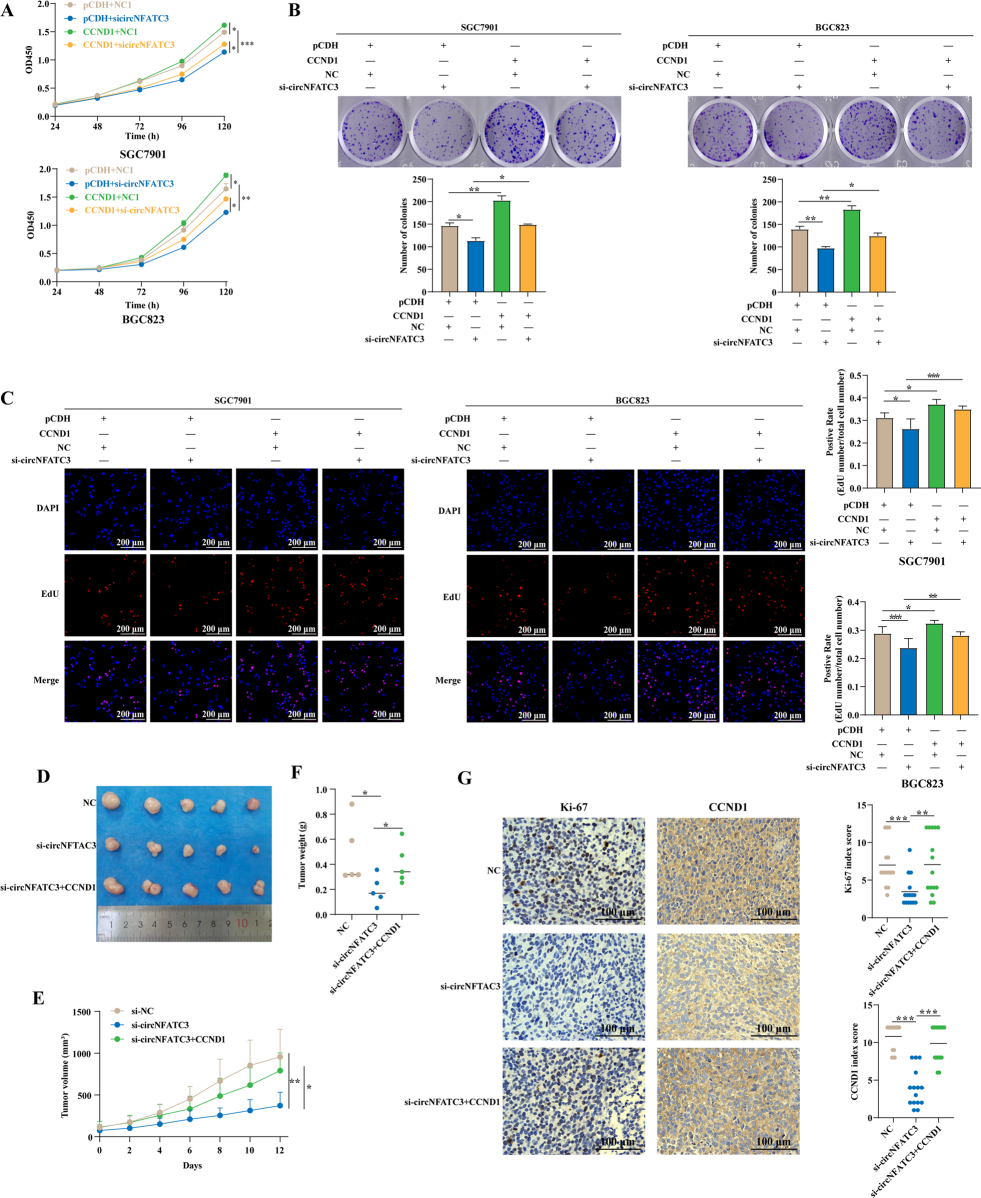
CircNFATC3 promotes the proliferation of GC via regulating CCND1. **A−C** CCK-8 (**A**), plate colony formation (**B**), and EdU assays (**C**) of SGC7901 and BGC823 cells transfected with blank vector or CCND1 overexpression plasmids and co-transfected with NC or si-circNFATC3 as indicated. **D−F** CCND1 lentivirus or si-circNFATC3 modified by cholesterol were injected into the xenograft tumor derived from HGC-27 cells. Tumor images (**D**), growth curves (**E**) and weights (**F**) were obtained from xenograft tumors derived from HGC-27 cells treated with cholesterol modified siRNAs targeting circNFATC3 or combined with CCND1 lentivirus. **G** Representative images of Ki-67 and CCND1 expression evaluated by IHC in xenografted tumor tissues. One-way ANOVA (**A−C**), the Student’s t test (**E**, **F**) and Mann–Whitney U test (**G**) were used to calculate p-values. *P < 0.05, **P < 0.01, ***P < 0.001

We have demonstrated that circNFATC3 regulates CCND1 expression in GC, so we additionally examined whether circNFATC3 can promote GC cell proliferation via this regulation. We firstly evaluated the role of CCND1 in GC proliferation and SGC7901 and BGC823 cell viability were significantly inhibited following a CCND1 knockdown (Additional file 2: Fig. S13A). Moreover, colony formation and EdU assays were consistent with these results (Additional file 2: Fig. S13B, C). CCND1 overexpression could also reverse the decrease of CCND1 caused by si-circNFATC3 (Additional file 2: Fig. S14) and rescued decreased proliferation caused by si-circNFATC3 (Fig. 7A–C). Moreover, in vivo rescue experiments based on HGC-27 constructed showed that lentivirus-mediated CCND1 overexpression significantly rescued the inhibitory effect of cholesterol modified si-circNFATC3 treatment on the subcutaneous tumors (Fig. 7D–F and Additional file 2: Fig. S15). In addition, expression of IGF2BP3-CCND1 axis and Ki-67 were downregulated in cholesterol modified si-circNFATC3-treated tumors, while lentivirus-mediated overexpression of CCND1 rescued the expression of CCND1 and Ki-67 (Fig. 7G and Additional file 2: Fig. S16). Taken together, these results indicated that circNFATC3 promoted the proliferation of GC via regulating the CCND1 pathway.

## Discussion

Previous studies have shown that IGF2BP3 levels are abnormally high in a variety of cancers and acts as an oncogene in various tumors, including GC [15–18, 31–33]. Usually, IGF2BP3 is an RBP and has been shown to affect tumor progression by binding to mRNA and affecting mRNA cleavage, translation and stability [8, 34, 35]. Additionally, there is evidence that IGF2BP3 can interact with non-coding RNAs including miRNAs, lncRNAs and circRNAs to affect tumor progression [12, 14, 36]. Especially, there is growing evidence that circRNAs can regulate the biological processes of numerous cancers through IGF2BP3 interactions [37, 38]. In GC, circFNDC3B cooperated with IGF2BP3 to enhance the expression of CD44, thus promoting GC invasion and metastasis [17]. Moreover, our previous study demonstrated that circTNPO3-IGF2BP3 or circARID1A-IGF2BP3 interaction contributed to GC tumorigenesis or progression [15, 18]. However, the interaction between IGF2BP3 and circRNAs is far from elucidated and the mechanisms for the action of circRNA-IGF2BP3 can lead to the identification of additional circRNAs that are linked with GC.

Here, we identified circNFATC3 that bound to the IGF2BP3 protein, and was upregulated in GC tissues compared with non-tumor tissues and was positively associated with tumor volume. Functional studies demonstrated that a circNFATC3 knockdown inhibited GC cell proliferation in vivo and in vitro. CircNFATC3 as a newly discovered circRNA has been reported to inhibit hepatocellular carcinoma progression [39] and promote the malignant phenotype of breast and ovarian cancer cells [40]. These data suggested that the functions of circNFATC3 are diverse and may play numerous roles in different tumors.

We tried to construct a circNFATC3 overexpression vector based on the pLC5-ciR, pcDNA3.1(+) circRNA Mini Vector and Pcd-ciR plasmid, commercial vector used for circRNA overexpression. No matter how we changed the transfection conditions, circNFATC3 was not successfully overexpressed in GC cell lines, while the linear sequence of circNFATC3 was elevated, which demonstrated that the cyclization of circNFATC3 was inhibited in GC cells. A previous study has shown that the biogenesis of exon-derived circRNAs required coordination between intronic repeats and exons and the sequence or length of exons is also a major factor affecting exon circularization. In addition, short intronic repeats are required for some exon circularization [41]. To test whether the circNFATC3 sequence affects the cyclization of GC cells, we constructed another 2 circRNAs overexpression vectors (pLC5-ciR-circPDHK1 and pCD-ciR-circTNPO3) and successfully overexpressed both circPDHK1 and circTNPO3. These results revealed that the popular strategy for overexpressing circRNA based on circularizing sequence flanked by canonical splice sites wasn’t applicable to circNFATC3 because of its base sequence. To date, there are other strategies to promote circRNA cyclization, such as overexpressing specific whole RBP or RNA binding motifs that can bind to the introns flanking circRNA forming exons to promote circrNA biogenesis[42–44]. In addition, circRNAs can be engineered in vitro through ribozymatic methods using self-splicing introns [45, 46]. However, above strategies have certain limitations. Firstly, RBP binding sites are required for the sequence of circRNAs. Secondly, some by-products may be produced and affect the function of circRNA. Thirdly, the purification and output of circRNAs engineered in vitro are obstacles that must be overcome. Therefore, the strategy of in vitro synthesis or promotion of cyclization may not be applicable to circNFATC3 because of the specificity of the sequence. In the future, we will further explore overexpression strategies of circNFATC3.

CircRNA-protein interactions are one of the ways that circRNAs exert their biological functions. Here, we found that circNFATC3 was primarily localized in the GC cell cytoplasm and could interact with both the N- and C-terminal regions of IGF2BP3. Interestingly, IGF2BP3 protein but not mRNA expression was significantly downregulated following circNFATC3 knockdown. Moreover, circNFATC3 knockdown in GC cells induced IGF2BP3 degradation through the ubiquitin–proteasome pathway. Similar to our results, previous studies have indicated that other circRNAs could also regulate protein expression. For example, circPFKFB4 inhibits DDB2 degradation in breast cancer [47] and circ_0006156 binds and stabilizes S100A9 in prostate cancer [48]. These data indicated a role for circRNAs in the post-transcriptional regulation of gene expression.

Ubiquitination is a post-translational modification that mediates protein degradation through the proteasome, thereby influencing tumor progression [49, 50]. E3 ligase enzymes play prominent roles in these ubiquitination processes. For example, FBXO22 can activate PD-L1 ubiquitination and degradation, thereby increasing the sensitivity of non-small cell lung cancer cells to DNA damage therapies [51]. In the present study, we found that TRIM25 not only inversely regulated the expression of the IGF2BP3 protein, but also interacted with IGF2BP3. Moreover, knockdown of TRIM25 could rescue the downregulation of IGF2BP3 caused by a circNFATC3 knockdown. Functionally, overexpression of IGF2BP3 partially rescued the inhibition of the proliferative capacity caused by circNFATC3 knockdown in GC cells. These results revealed that circNFATC3 enhances the stability of IGF2BP3 by preventing TRIM25-mediated ubiquitination and the interaction between circNFATC3 and IGF2BP3 might be the key to explain the ability of circNFATC3 to promote GC proliferation. Consistent with our results, circNEIL3 binds to IGF2BP3 and inhibits the ubiquitination of IGF2BP3 by E3 ubiquitin ligase HECTD4, thereby inhibiting the degradation of IGF2BP3 by the proteasome and promoting the proliferation and metastasis of glioma cells [37]. In hepatocellular carcinoma, circRNA-SORE binds the major oncogenic protein YBX1 in the cytoplasm thereby preventing the nuclear interaction of YBX1 with the E3 ubiquitin ligase PRP19 thereby reducing YBX1 levels [52].

IGF2BP3 is an RBP and can affect tumor progression by targeting mRNA for MMP1, CD44, CDK164, ABCG2, IGF2 and other genes [34]. In our study, we confirmed that CCND1 mRNA interacts with IGF2BP3 and also is a target of the circNFATC3-IGF2BP3 axis. CCND1 is a D-cyclin and serves as an effector gene promoting tumor progression in a variety of cancers including GC [26, 27, 53]. Here, we found that circNFATC3-IGF2BP3 axis enhanced CCND1 mRNA stability that promoted GC cell proliferation. CCND1 overexpression could rescue the effects of a circNFATC3 knockdown and GC proliferation in vitro and in vivo. Taken together, these results suggested that circNFATC3-IGF2BP3 axis promotes GC cell proliferation by regulating the stability of CCND1 mRNA.

Nucleic acid therapy can effectively regulate coding and non-coding target genes with high specificity and is attracting special attention. At present, there are increasing numbers of nucleic acid drugs that are approved or are in clinical trials including siRNA drugs and antisense oligonucleotides [54, 55]. In our study, circNFATC3 was significantly overexpressed in GC tissues and promoted the proliferation of GC cells in vivo and in vitro. Our results suggested that circNFATC3 is an oncogene in GC and might be a potential therapeutic target for GC.

## Conclusions

**Fig. 8:**
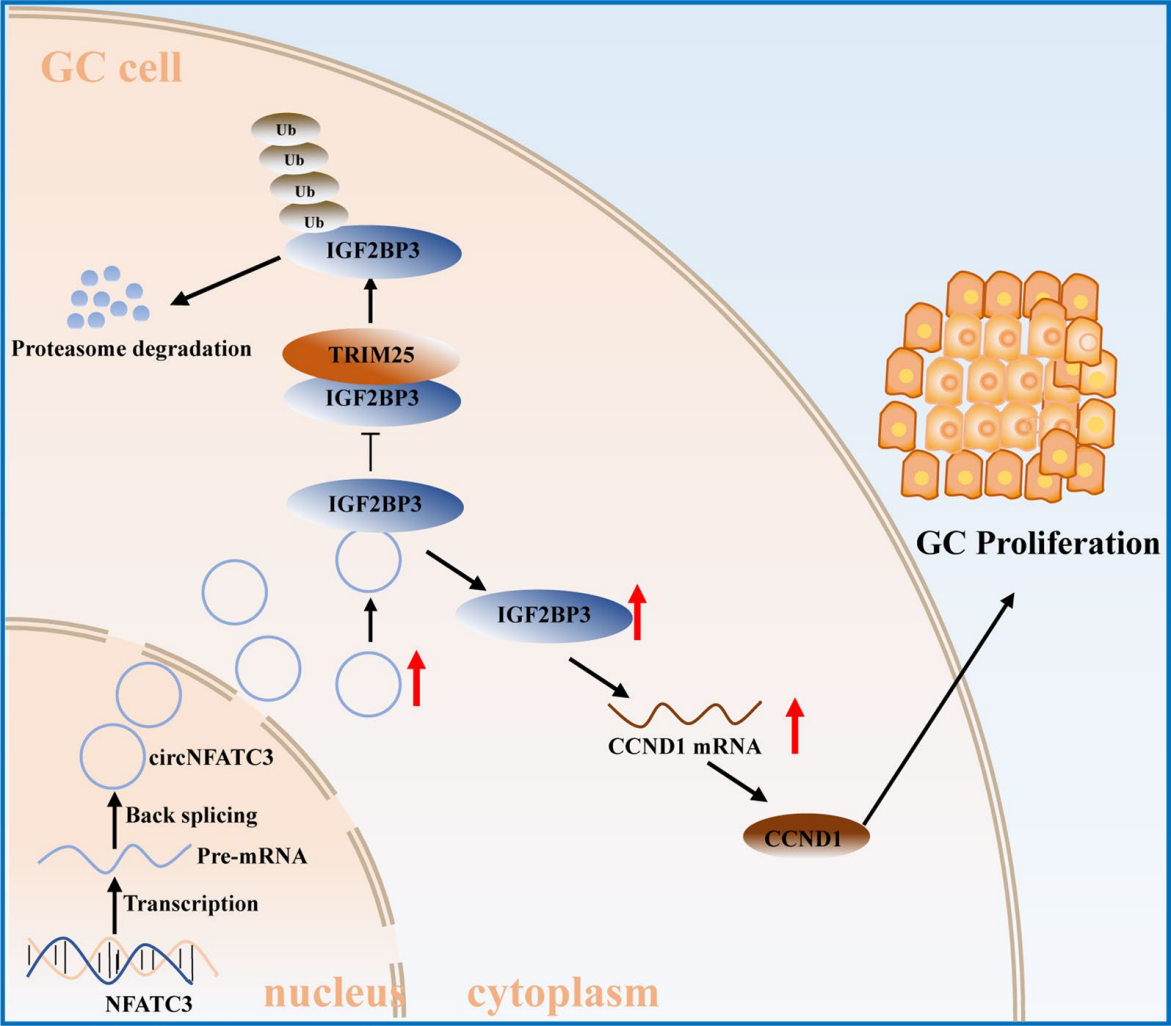
A schematic illustration of the molecular mechanism of circNFATC3 in regulating the proliferation of GC. CircNFATC3 expression is up-regulated in GC. CircNFATC3 enhances the stability of IGF2BP3 by preventing TRIM25-mediated ubiquitination, subsequently elevating the stability of CCND1 and promoting GC proliferation

Here, we identified an IGF2BP3-binding circRNA, circNFATC3, that was significantly overexpressed in GC tissues and was positively associated with tumor volume. Functionally, circNFATC3 promoted the proliferation of GC cells in vivo and in vitro. Mechanistically, circNFATC3 bound to IGF2BP3 in the cytoplasm and enhanced the stability of IGF2BP3 by preventing ubiquitin E3 ligase TRIM25-mediated ubiquitination, thereby enhancing the regulatory axis of IGF2BP3-CCND1 and promoting CCND1 mRNA stability. Our results indicated that circNFATC3 may be a novel therapeutic target for GC (Fig. 8).

## Supplementary Information

**Additional file 1: Table S1.** Characteristics of GC patients for circNFATC3 measurement. **Table S2.** Probes for RNA-FISH. **Table S3.** Primers for amplification of indicated circRNAs and genes. **Table S4.** Characteristics of GC patients for TMA. **Table S5.** Probes targeting circNFATC3 for TMA. **Table S6.** SiRNAs sequences. **Table S7.** Probes for RNA pull-down. **Table S8.** CircRNAs interacting with IGF2BP3 in SGC7901 cells (Top 50). **Table S9.** Upregulated genes in STAD of the TCGA database (Log2(Fold Change)＞2). **Table S10.** mRNAs interacting with IGF2BP3 in SGC7901 cells evaluated by RIP-seq. **Table S11.** Downregulated genes evaluated by RNA-seq in SGC7901 cells following IGF2BP3 knockdown.

**Additional file 2: Fig. S1. **The expression of circELK4, circARID1A, circFNDC3B, and circNFATC3 in GC tissues and corresponding adjacent tissues (n=16). The *P*-values were calculated using the Mann-Whitney U test. ns, not significant, **P < 0.01. **Fig. S2.** CircNFATC3 sequencing results of GC tissues and the corresponding adjacent tissues, as well as results from SGC7901 and BGC823 cell lines. **Fig. S3. **Effect of circNFATC3 knockdown on the expression levels of circNFATC3 or NFATC3 mRNA in GC cells. **A** The interference efficiency of si-circNFATC3-1 and si-circNFATC3-2 in SGC7901 and BGC823 cells. **B** The expression of NFATC3 mRNA in SGC7901 and BGC823 cells treated with circNFATC3 knockdown by siRNAs. The *P*-values were calculated using Student’s t test. ns, not significant, *P < 0.05, **P < 0.01. **Fig. S4. **Knockdown efficiency of IGF2BP3 and circNFATC3 in SGC7901 and BGC823 cells. **A** The expression of IGF2BP3 in SGC7901 and BGC823 cells after IGF2BP3 knockdown by siRNA. **B** The expression of circNFATC3 in SGC7901 and BGC823 cells after circNFATC3 knockdown by siRNA. The *P*-values were calculated using Student’s t test. **P < 0.01, ***P < 0.001. **Fig. S5. **Effect of circNFATC3 knockdown by si-circNFATC3-1 on proliferation of GC cells. **A** The viability of SGC7901 and BGC823 cells after transfected with si-circNFATC3-1. **B** Plate colony formation of SGC7901 and BGC823 cells after transfected with si-circNFATC3-1.** C** The EdU assay of SGC7901 and BGC823 cells after transfected with si-circNFATC3-1. The *P*-values were calculated using Student’s t test. *P < 0.05, **P < 0.01, ***P < 0.001. **Fig. S6. **Effect of circNFATC3 stable knockdown on the proliferation of SGC7901 cells. (A) Efficiency of lentivirus sh-circNFATC3 transduction in SGC7901 cells. (B) Cell viability of SGC7901 cell following circNFATC3 stable knockdown. (C) Plate colony formation assay of SGC7901 cells with circNFATC3 stable knockdown. (D) The EdU assay of SGC7901 cells with circNFATC3 stable knockdown. The *P*-values were calculated using Student’s t test. **P < 0.01, ***P < 0.001. **Fig. S7. **Overexpression of circNFATC3 in GC cells by using different vectors. **A-B **Plasmid profile of pLC5-ciR (**A**) and overexpression efficiency of circNFATC3 in SGC7901 and BGC823 cells, as well as linear product of circNFATC3 in SGC7901 cells (**B**).** C-D **Plasmid profile of pcDNA3.1(+) CircRNA Mini Vector (**C**) and overexpression efficiency of circNFATC3 in SGC7901 and BGC823 cells, as well as linear product of circNFATC3 in BGC823 cells (**D**).** E-F **Plasmid profile of pCD-ciR (**E**) and overexpression efficiency of circNFATC3 or linear product in SGC7901 cells (**F**). **G **The overexpression of pLC5-ciR-circPDHK1 in SGC7901 and BGC823 cells. **H** The overexpression of pCD-ciR-circTNPO3 in SGC7901 and BGC823 cells. The *P*-values were calculated using Student’s t test. ns, not significant, *P < 0.05, **P < 0.01, ***P < 0.001. **Fig. S8. **Regulation of circNFATC3 and IGF2BP3 to each other in GC cells. **A-B** The expression of IGF2BP3 at RNA (**A**) and protein (**B**) level in SGC7901 and BGC823 cells transfected with si-IGF2BP3. **C** The expression of circNFATC3 in SGC7901 and BGC823 cells after IGF2BP3 knockdown. **D-E** The expression of IGF2BP3 at RNA (**D**) and protein (**E**) level in SGC7901 and BGC823 cells transfected with pCDH-IGF2BP3. **F **The expression of circNFATC3 in SGC7901 and BGC823 cells after IGF2BP3 overexpression. **G** The expression of IGF2BP3 at RNA level in SGC7901 and BGC823 cells after circNFATC3 knockdown. The *P*-values were calculated using Student’s t test. ns, not significant, *P < 0.05, **P < 0.01, ***P < 0.001. **Fig. S9.** Quantitative analysis of IGF2BP3 protein stability after circNFATC3 knockdown in SGC7901 and BGC823. **Fig. S10. **Expression of LC3 in SGC7901 and BGC823 cells after circNFATC3 knockdown. **Fig. S11. **Overexpression efficiency of TRIM25 (HA-tagged) and IGF2BP3 truncations (GFP-tagged) in SGC7901 and BGC823 cells. **Fig. S12. **Expression of RCC2, ARL6IP1, CCND1, and MUC13 mRNA in SGC7901 and BGC823 cells treated with si-IGF2BP3, pCDH-IGF2BP3, or si-circNFATC3. **A** Expression of RCC2, ARL6IP1, CCND1, and MUC13 mRNA in SGC7901 and BGC823 after IGF2BP3 knockdown. **B** Expression of RCC2, ARL6IP1, CCND1, and MUC13 mRNA in SGC7901 and BGC823 treated with IGF2BP3 overexpression.** C** Expression of RCC2, ARL6IP1, CCND1, and MUC13 mRNA in SGC7901 and BGC823 after circNFATC3 knockdown. The *P*-values were calculated using Student’s t test. ns, not significant, *P < 0.05, **P < 0.01，***P < 0.001. **Fig. S13. **The proliferation of SGC7901 and BGC823 cells after CCND1 knockdown. **A** The viability of SGC7901 and BGC823 cells transfected with si-CCND1. **B** Plate colony formation of SGC7901 and BGC823 cells transfected with si-CCND1.** C** The EdU assay of SGC7901 and BGC823 cells transfected with si-CCND1. The *P*-values were calculated using Student’s t test. *P < 0.05, **P < 0.01，***P < 0.001. **Fig. S14. **Expression of CCND1 protein in SGC7901 and BGC823 cells transfected with blank vector or pCDH-CCND1 and co-transfected with NC or circNFATC3 siRNA. **Fig. S15. **Expression of circNFATC3 and CCND1 in xenograft tumor tissues. **A** Expression of circNFATC3 in xenograft tumor tissues treated with lentivirus containing CCND1 or cholesterol modified si-circNFATC3.** B** Expression of CCND1 in xenograft tumor tissues treated with lentivirus containing CCND1 or cholesterol modified si-circNFATC3. The *P*-values were calculated using Student’s t test. ns, not significant, *P < 0.05, ***P < 0.001. **Fig. S16. **Representative images of IGF2BP3 expression evaluated by IHC in xenograft tumor tissues. Three different visual fields were randomly selected for each slice. The *P*-values were calculated using Mann-Whitney U test. ***P < 0.001.

## Data Availability

All data used in this work can be acquired from the corresponding author upon reasonable request.

## Abbreviations

GC: Gastric cancer
IGF2BP3: Insulin like growth factor II mRNA binding protein 3
RBP: RNA binding protein
circRNAs: Circular RNAs
RIP-seq: RNA immunoprecipitation and sequencing
qRT-PCR: Quantitative real time-polymerase chain reaction
RNA-FISH: RNA fluorescence in situ hybridization
TMA: Tissue microarray
ISH: In situ hybridization
RIP: RNA-binding protein immunoprecipitation
IF: Immunofluorescence
IP: Immunoprecipitation
CCND1: Cyclin D1
TRIM25: Tripartite motif-containing 25
CCK-8: Cell counting Kit-8
EdU: 5-Ethynyl-2´-deoxyuridine
IHC: Immunohistochemistry
CHX: Cycloheximide
MG132: Proteasome inhibitor
TCGA: The cancer genome atlas
TIMER: Tumor Immune Estimation Resource
