# Supplementary material for: CircNFATC3 promotes the proliferation of gastric cancer through binding to IGF2BP3 and restricting its ubiquitination to enhance CCND1 mRNA stability
Source: J Transl Med. 2023 Jun 20;21:402. doi: 10.1186/s12967-023-04235-y (PMC10280940; doi:10.1186/s12967-023-04235-y)
Supplement: Supplementary file 2 — Additional file 2: Fig. S1. The expression of circELK4, circARID1A, circFNDC3B, and circNFATC3 in GC tissues and corresponding adjacent tissues (n=16). The P-values were calculated using the Mann-Whitney U test. ns, not significant, **P < 0.01. Fig. S2. CircNFATC3 sequencing results of GC tissues and the corresponding adjacent tissues, as well as results from SGC7901 and BGC823 cell lines. Fig. S3. Effect of circNFATC3 knockdown on the expression levels of circNFATC3 or NFATC3 mRNA in GC cells. A The interference efficiency of si-circNFATC3-1 and si-circNFATC3-2 in SGC7901 and BGC823 cells. B The expression of NFATC3 mRNA in SGC7901 and BGC823 cells treated with circNFATC3 knockdown by siRNAs. The P-values were calculated using Student’s t test. ns, not significant, *P < 0.05, **P < 0.01. Fig. S4. Knockdown efficiency of IGF2BP3 and circNFATC3 in SGC7901 and BGC823 cells. A The expression of IGF2BP3 in SGC7901 and BGC823 cells after IGF2BP3 knockdown by siRNA. B The expression of circNFATC3 in SGC7901 and BGC823 cells after circNFATC3 knockdown by siRNA. The P-values were calculated using Student’s t test. **P < 0.01, ***P < 0.001. Fig. S5. Effect of circNFATC3 knockdown by si-circNFATC3-1 on proliferation of GC cells. A The viability of SGC7901 and BGC823 cells after transfected with si-circNFATC3-1. B Plate colony formation of SGC7901 and BGC823 cells after transfected with si-circNFATC3-1. C The EdU assay of SGC7901 and BGC823 cells after transfected with si-circNFATC3-1. The P-values were calculated using Student’s t test. *P < 0.05, **P < 0.01, ***P < 0.001. Fig. S6. Effect of circNFATC3 stable knockdown on the proliferation of SGC7901 cells. (A) Efficiency of lentivirus sh-circNFATC3 transduction in SGC7901 cells. (B) Cell viability of SGC7901 cell following circNFATC3 stable knockdown. (C) Plate colony formation assay of SGC7901 cells with circNFATC3 stable knockdown. (D) The EdU assay of SGC7901 cells with circNFATC3 stable knockdown. The P-values were calcu [file 12967_2023_4235_MOESM2_ESM.docx]

**Additional Figures**

**
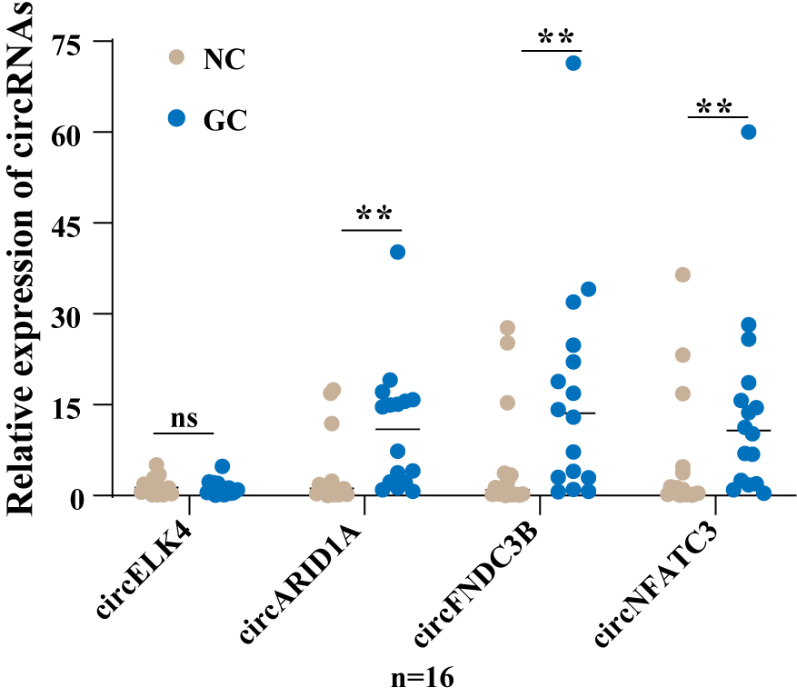
**

**Fig. S1** The expression of circELK4, circARID1A, circFNDC3B, and circNFATC3 in GC tissues and corresponding adjacent tissues (n=16). The *P*-values were calculated using the Mann-Whitney U test. ns, not significant, ***P* < 0.01.


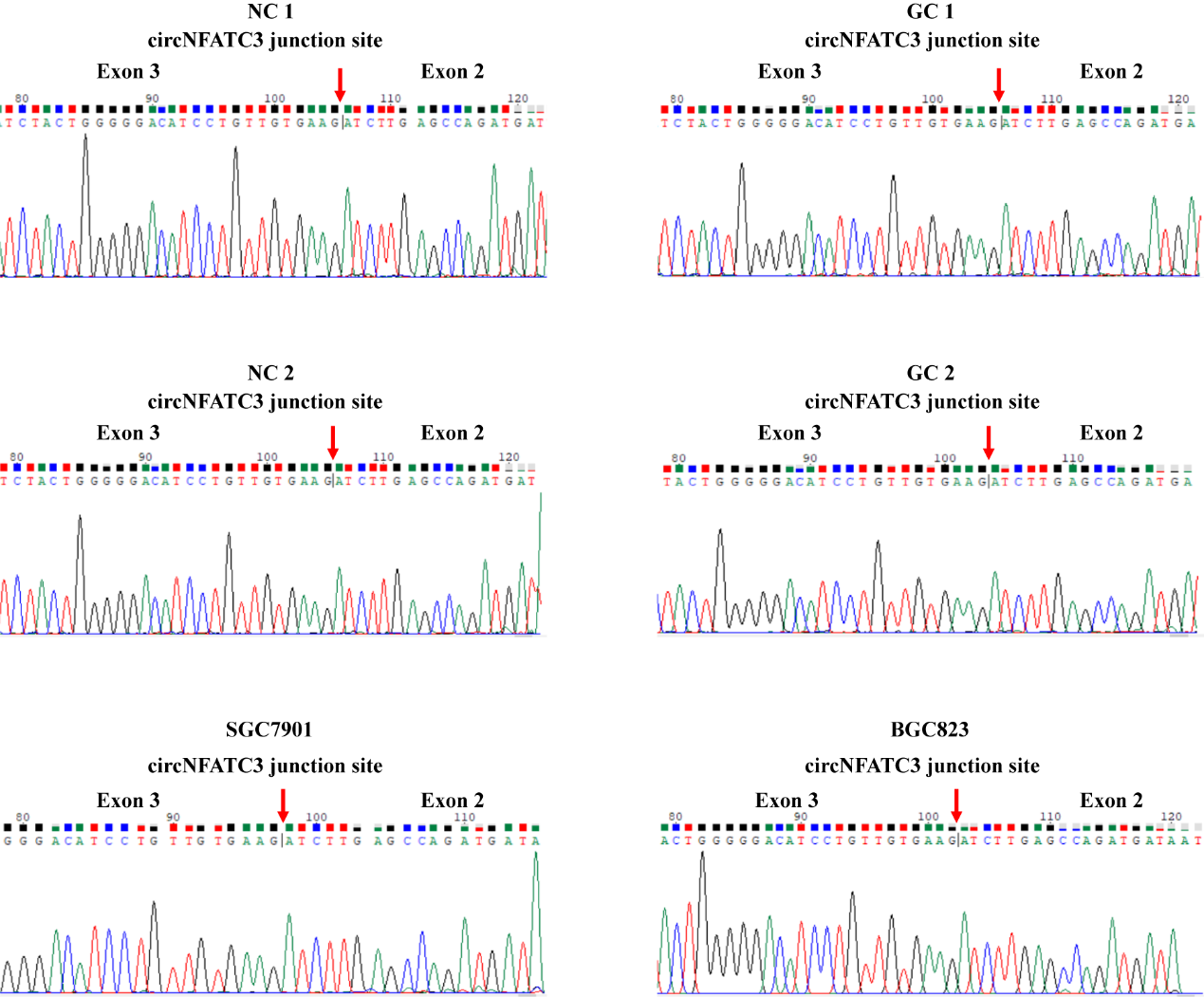


**Fig. S2** CircNFATC3 sequencing results of GC tissues and the corresponding adjacent tissues, as well as results from SGC7901 and BGC823 cell lines.

**
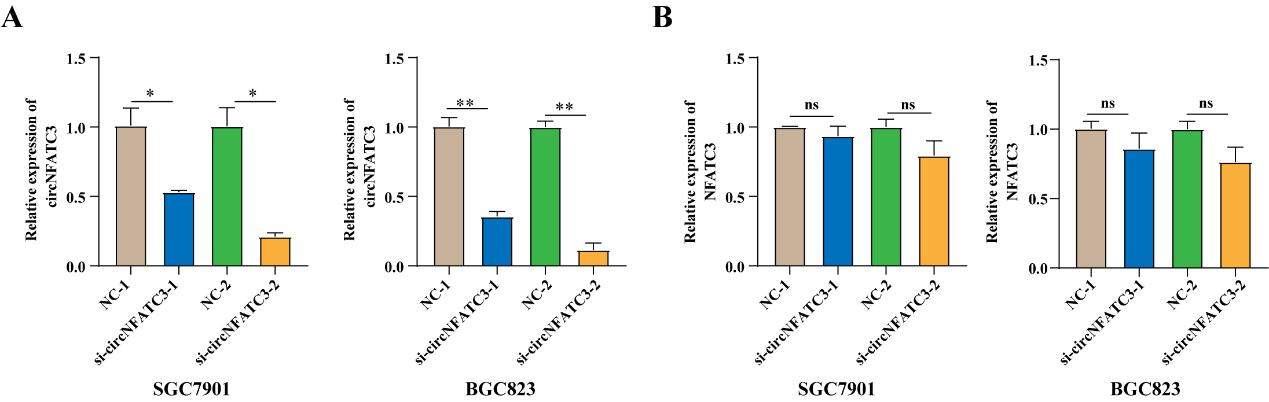
**

**Fig. S3** Effect of circNFATC3 knockdown on the expression levels of circNFATC3 or NFATC3 mRNA in GC cells. **A** The interference efficiency of si-circNFATC3-1 and si-circNFATC3-2 in SGC7901 and BGC823 cells. **B** The expression of NFATC3 mRNA in SGC7901 and BGC823 cells treated with circNFATC3 knockdown by siRNAs. The *P*-values were calculated using Student’s t test. ns, not significant, **P* < 0.05, ***P* < 0.01.

**
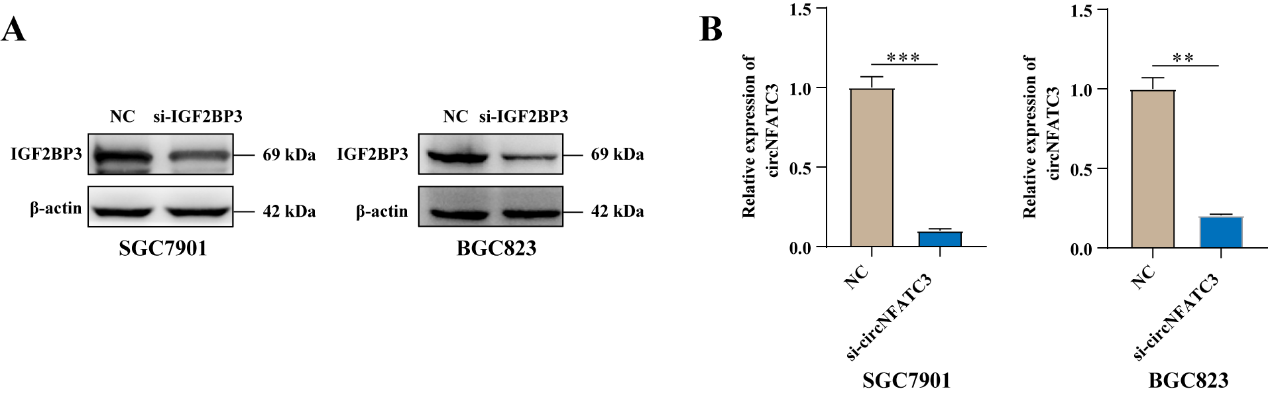
**

**Fig. S4** Knockdown efficiency of IGF2BP3 and circNFATC3 in SGC7901 and BGC823 cells. **A** The expression of IGF2BP3 in SGC7901 and BGC823 cells after IGF2BP3 knockdown by siRNA. **B** The expression of circNFATC3 in SGC7901 and BGC823 cells after circNFATC3 knockdown by siRNA. The *P*-values were calculated using Student’s t test. ***P* < 0.01, ****P* < 0.001.

**
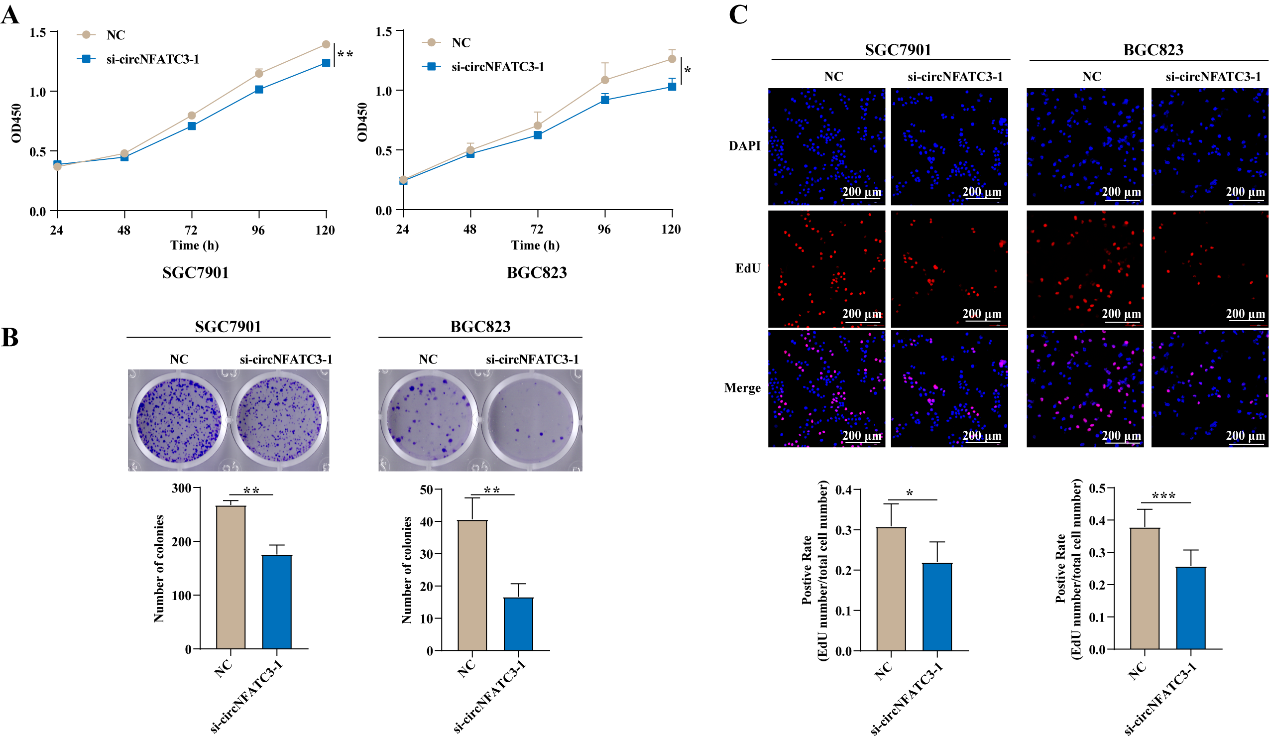
**

**Fig. S5** Effect of circNFATC3 knockdown by si-circNFATC3-1 on proliferation of GC cells. **A** The viability of SGC7901 and BGC823 cells after transfected with si-circNFATC3-1. **B** Plate colony formation of SGC7901 and BGC823 cells after transfected with si-circNFATC3-1. **C** The EdU assay of SGC7901 and BGC823 cells after transfected with si-circNFATC3-1. The *P*-values were calculated using Student’s t test. **P* < 0.05, ***P* < 0.01, ****P* < 0.001.

**
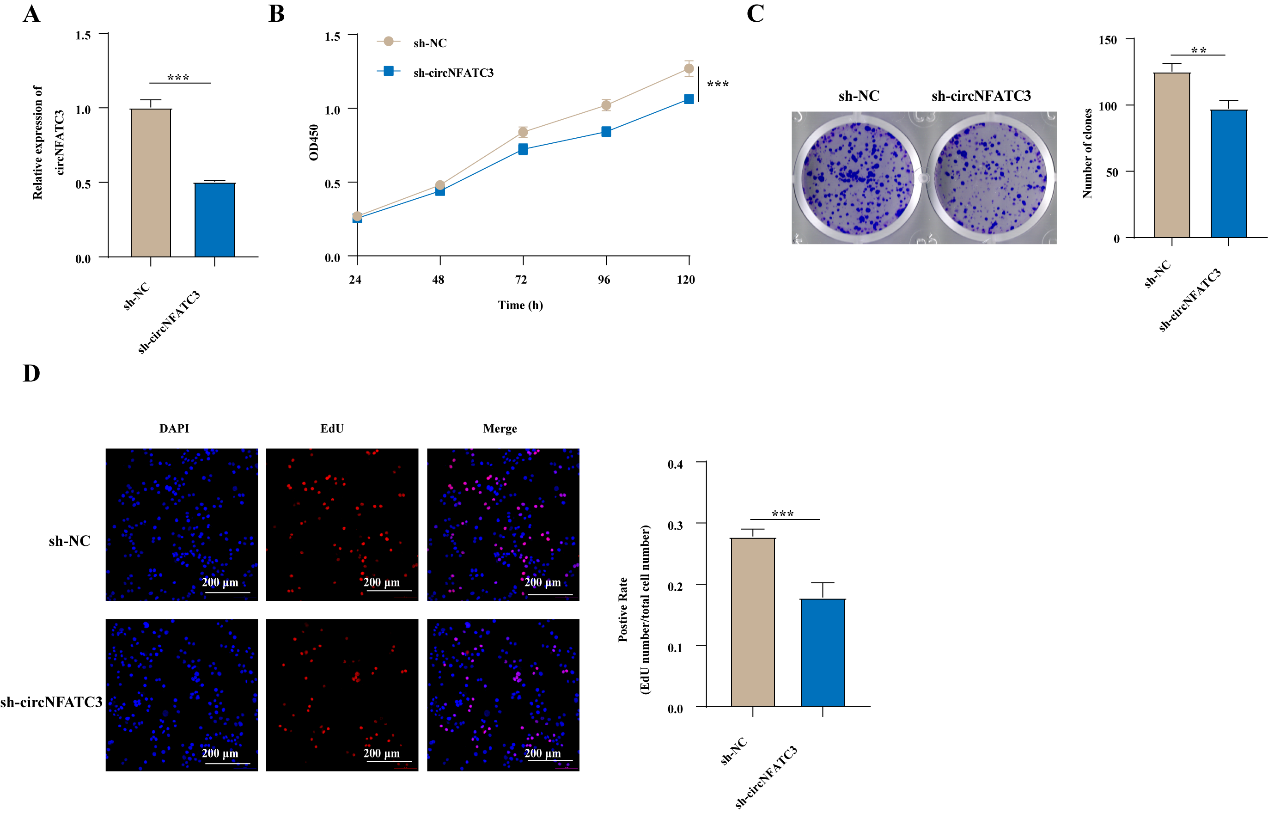
**

**Fig. S6** Effect of circNFATC3 stable knockdown on the proliferation of SGC7901 cells. (A) Efficiency of lentivirus sh-circNFATC3 transduction in SGC7901 cells. (B) Cell viability of SGC7901 cell following circNFATC3 stable knockdown. (C) Plate colony formation assay of SGC7901 cells with circNFATC3 stable knockdown. (D) The EdU assay of SGC7901 cells with circNFATC3 stable knockdown. The *P*-values were calculated using Student’s t test. ***P* < 0.01, ****P* < 0.001.

**
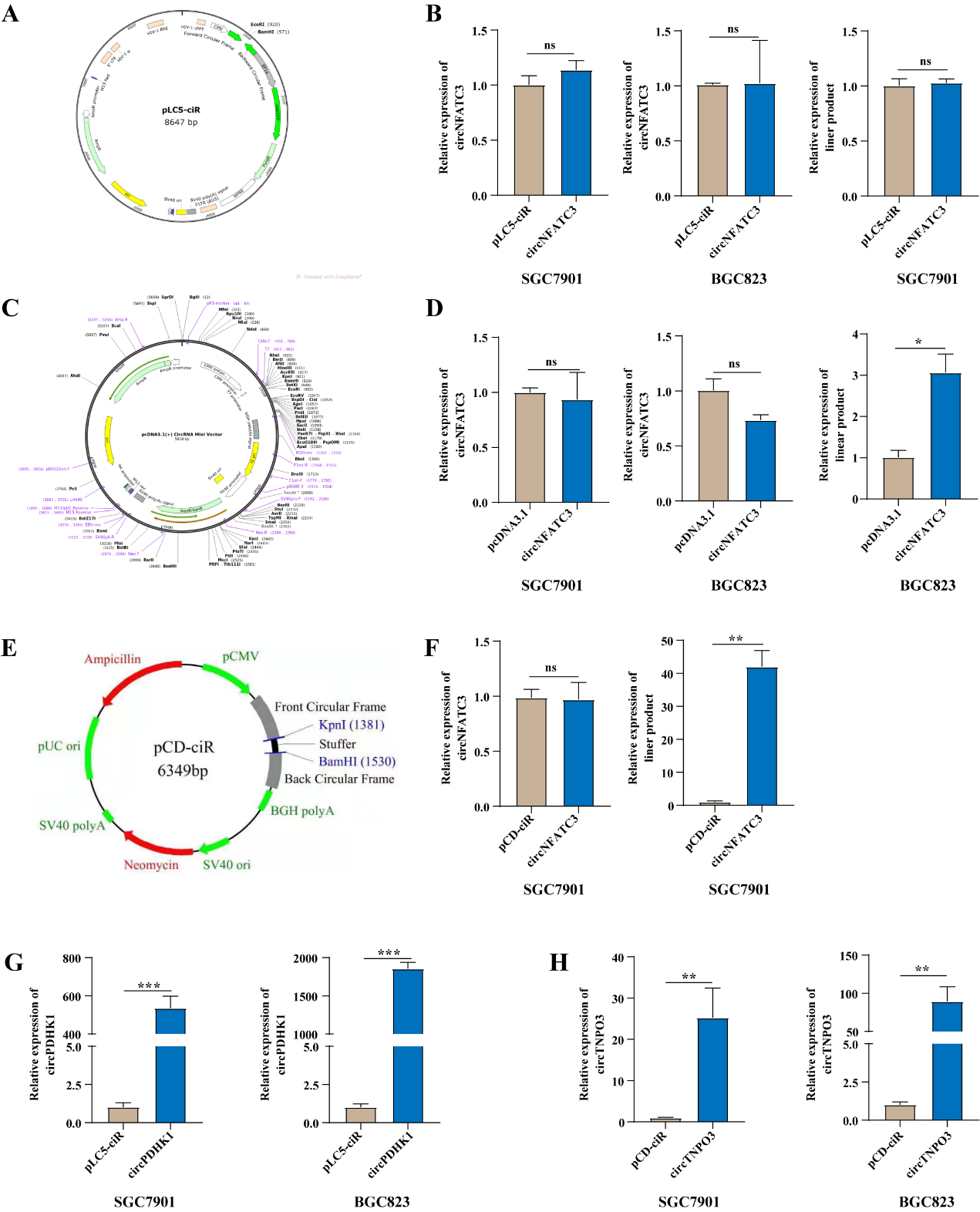
**

**Fig. S7** Overexpression of circNFATC3 in GC cells by using different vectors. **A-B** Plasmid profile of pLC5-ciR (**A**) and overexpression efficiency of circNFATC3 in SGC7901 and BGC823 cells, as well as linear product of circNFATC3 in SGC7901 cells (**B**). **C-D** Plasmid profile of pcDNA3.1(+) CircRNA Mini Vector (**C**) and overexpression efficiency of circNFATC3 in SGC7901 and BGC823 cells, as well as linear product of circNFATC3 in BGC823 cells (**D**). **E-F** Plasmid profile of pCD-ciR (**E**) and overexpression efficiency of circNFATC3 or linear product in SGC7901 cells (**F**). **G** The overexpression of pLC5-ciR-circPDHK1 in SGC7901 and BGC823 cells. **H** The overexpression of pCD-ciR-circTNPO3 in SGC7901 and BGC823 cells. The *P*-values were calculated using Student’s t test. ns, not significant, **P* < 0.05, ***P* < 0.01, ****P* < 0.001.

**
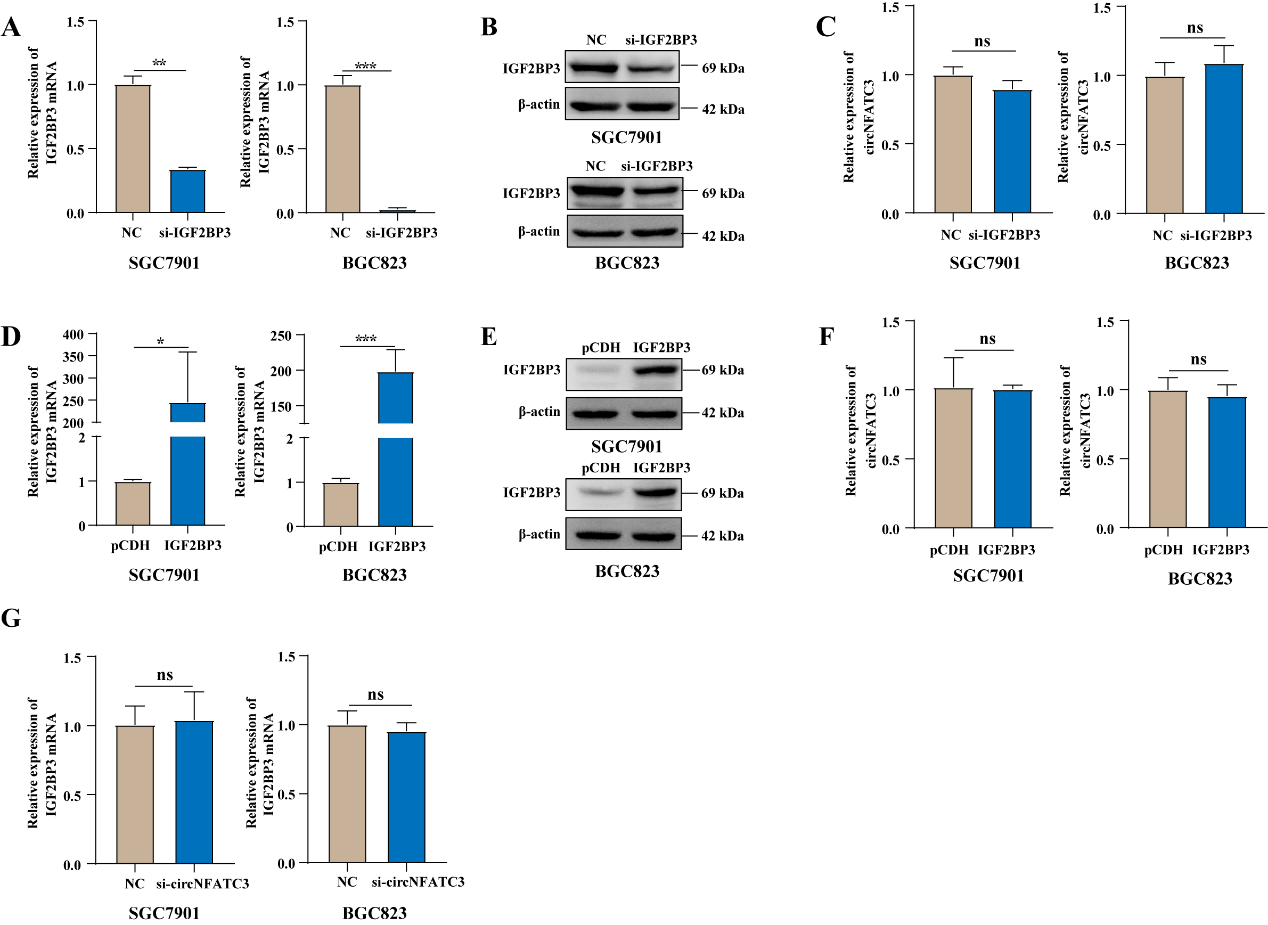
**

**Fig. S8** Regulation of circNFATC3 and IGF2BP3 to each other in GC cells. **A-B** The expression of IGF2BP3 at RNA (**A**) and protein (**B**) level in SGC7901 and BGC823 cells transfected with si-IGF2BP3. **C** The expression of circNFATC3 in SGC7901 and BGC823 cells after IGF2BP3 knockdown. **D-E** The expression of IGF2BP3 at RNA (**D**) and protein (**E**) level in SGC7901 and BGC823 cells transfected with pCDH-IGF2BP3. **F** The expression of circNFATC3 in SGC7901 and BGC823 cells after IGF2BP3 overexpression. **G** The expression of IGF2BP3 at RNA level in SGC7901 and BGC823 cells after circNFATC3 knockdown. The *P*-values were calculated using Student’s t test. ns, not significant, **P* < 0.05, ***P* < 0.01, ****P* < 0.001.


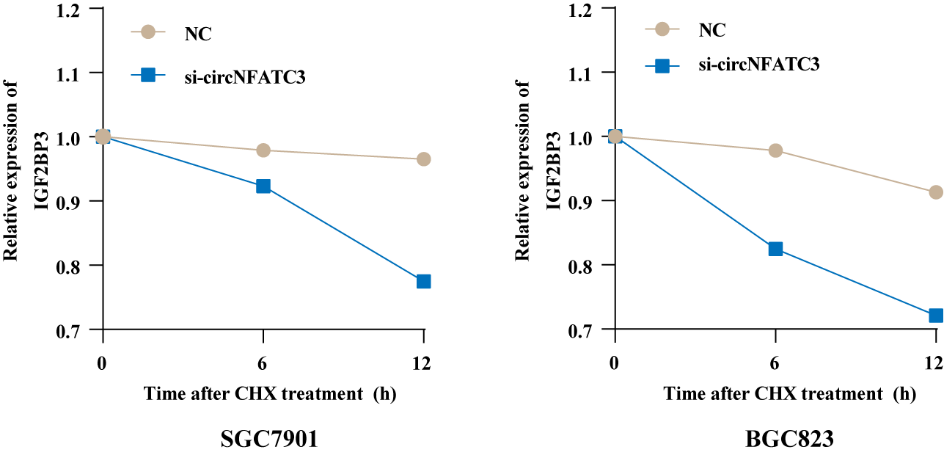


**Fig. S9** Quantitative analysis of IGF2BP3 protein stability after circNFATC3 knockdown in SGC7901 and BGC823.


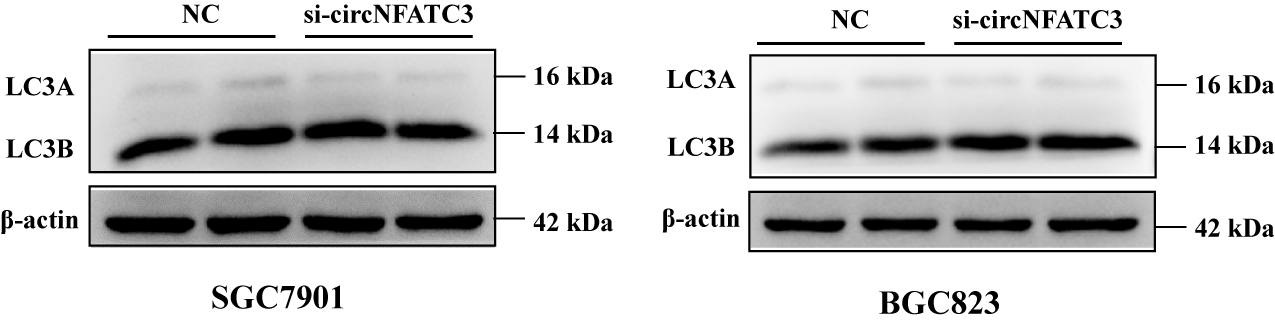


**Fig. S10** Expression of LC3 in SGC7901 and BGC823 cells after circNFATC3 knockdown.

**
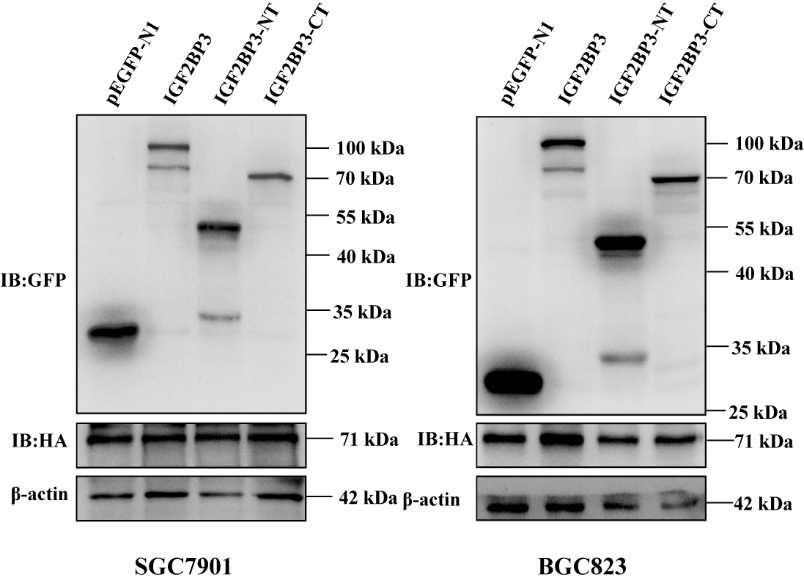
**

**Fig. S11** Overexpression efficiency of TRIM25 (HA-tagged) and IGF2BP3 truncations (GFP-tagged) in SGC7901 and BGC823 cells.


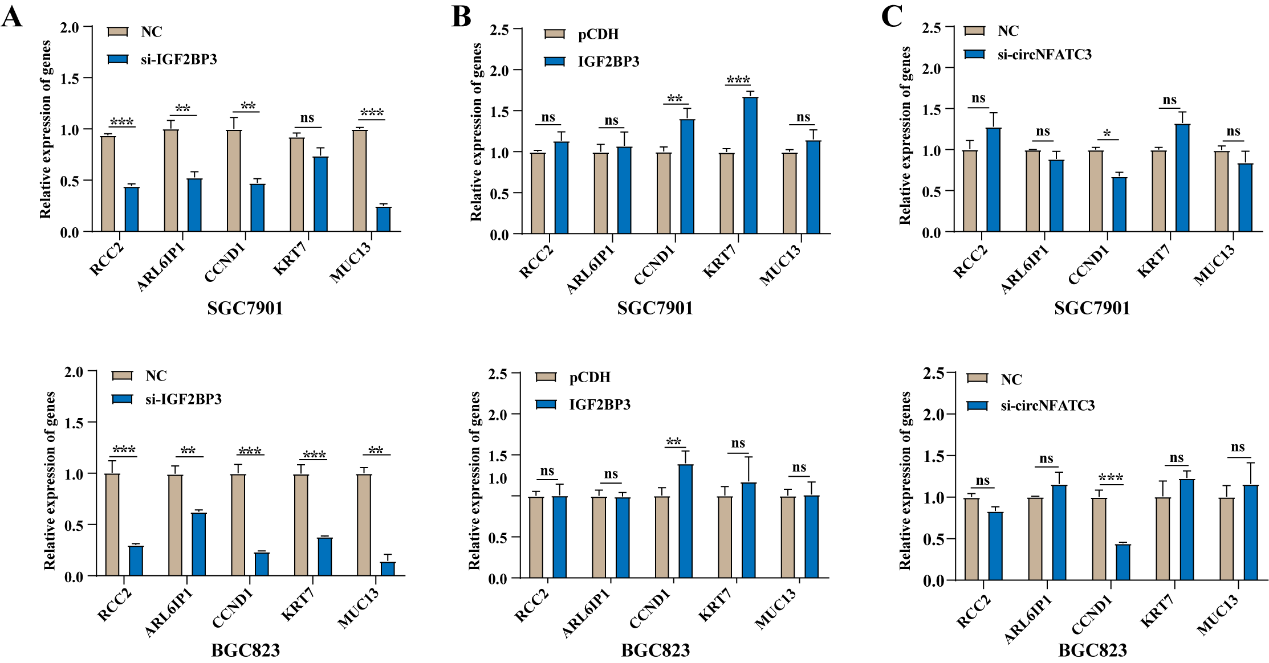


**Fig. S12** Expression of RCC2, ARL6IP1, CCND1, and MUC13 mRNA in SGC7901 and BGC823 cells treated with si-IGF2BP3, pCDH-IGF2BP3, or si-circNFATC3. **A** Expression of RCC2, ARL6IP1, CCND1, and MUC13 mRNA in SGC7901 and BGC823 after IGF2BP3 knockdown. **B** Expression of RCC2, ARL6IP1, CCND1, and MUC13 mRNA in SGC7901 and BGC823 treated with IGF2BP3 overexpression. **C** Expression of RCC2, ARL6IP1, CCND1, and MUC13 mRNA in SGC7901 and BGC823 after circNFATC3 knockdown. The *P*-values were calculated using Student’s t test. ns, not significant, **P* < 0.05, ***P* < 0.01，****P* < 0.001.


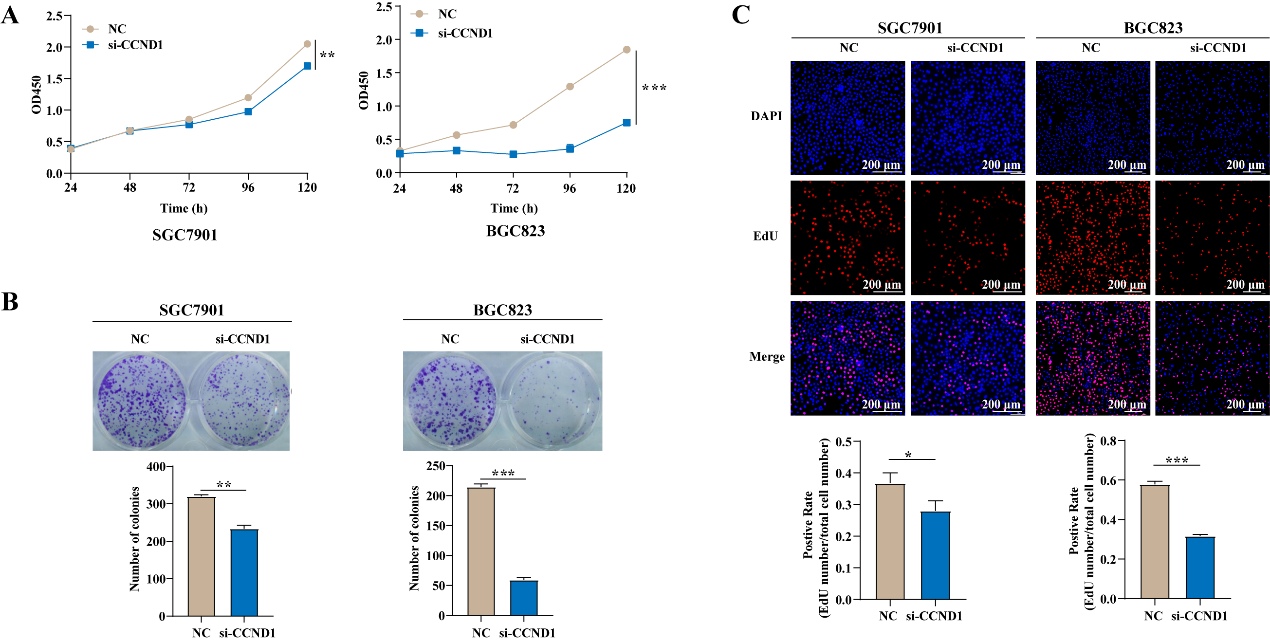


**Fig. S13** The proliferation of SGC7901 and BGC823 cells after CCND1 knockdown. **A** The viability of SGC7901 and BGC823 cells transfected with si-CCND1. **B** Plate colony formation of SGC7901 and BGC823 cells transfected with si-CCND1. **C** The EdU assay of SGC7901 and BGC823 cells transfected with si-CCND1. The *P*-values were calculated using Student’s t test. **P* < 0.05, ***P* < 0.01，****P* < 0.001.


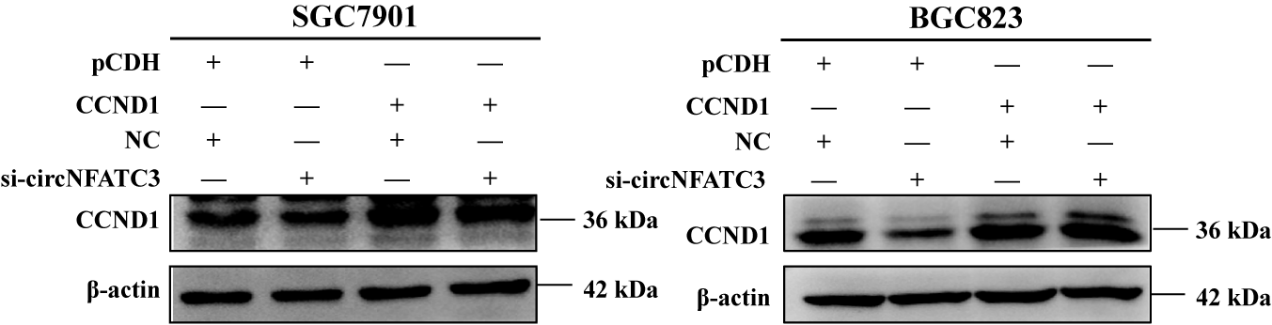


**Fig. S14** Expression of CCND1 protein in SGC7901 and BGC823 cells transfected with blank vector or pCDH-CCND1 and co-transfected with NC or circNFATC3 siRNA.


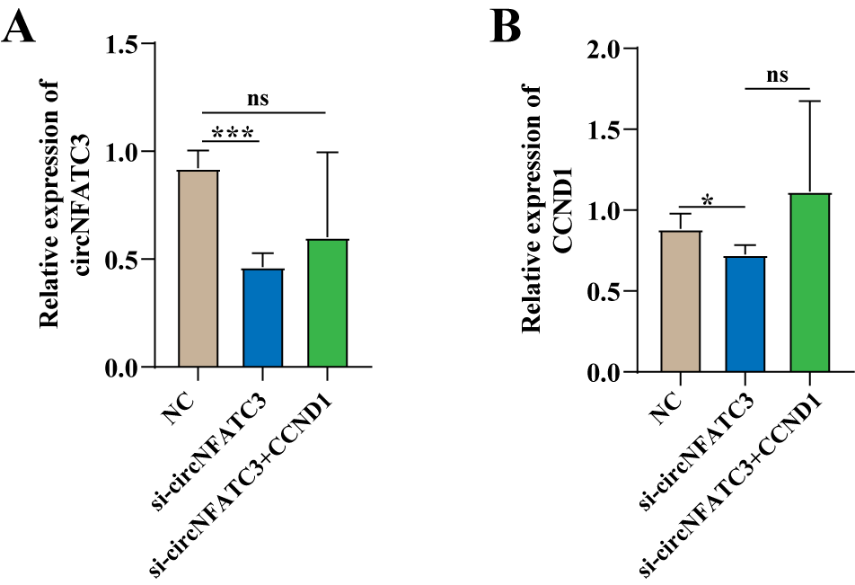


**Fig. S15** Expression of circNFATC3 and CCND1 in xenograft tumor tissues. **A** Expression of circNFATC3 in xenograft tumor tissues treated with lentivirus containing CCND1 or cholesterol modified si-circNFATC3. **B** Expression of CCND1 in xenograft tumor tissues treated with lentivirus containing CCND1 or cholesterol modified si-circNFATC3. The *P*-values were calculated using Student’s t test. ns, not significant, **P* < 0.05, ****P* < 0.001.


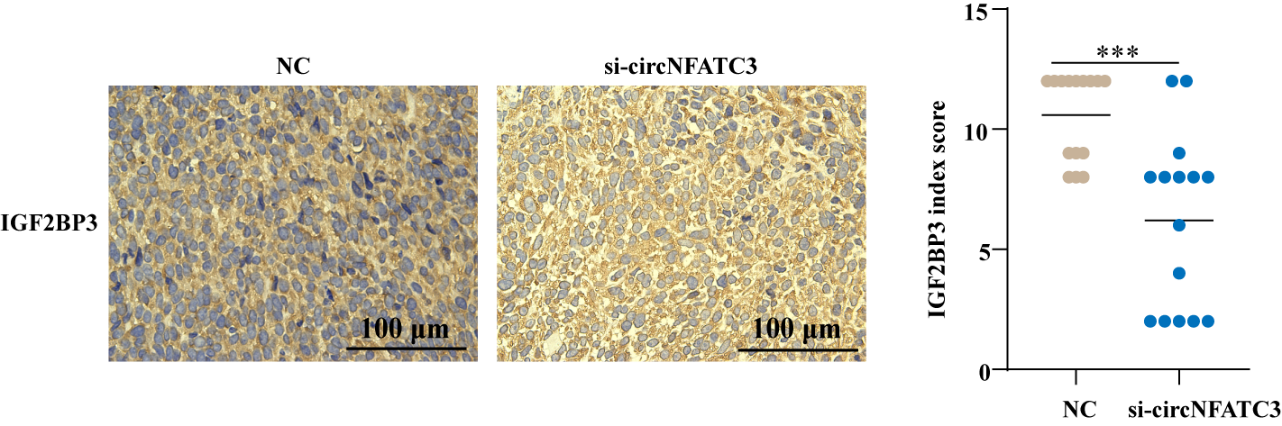


**Fig. S16** Representative images of IGF2BP3 expression evaluated by IHC in xenograft tumor tissues. Three different visual fields were randomly selected for each slice. The *P*-values were calculated using Mann-Whitney U test. ****P* < 0.001.
